# An Improved Search and Rescue Algorithm for Global Optimization and Blood Cell Image Segmentation

**DOI:** 10.3390/diagnostics13081422

**Published:** 2023-04-15

**Authors:** Essam H. Houssein, Gaber M. Mohamed, Nagwan Abdel Samee, Reem Alkanhel, Ibrahim A. Ibrahim, Yaser M. Wazery

**Affiliations:** 1 Faculty of Computers and Information, Minia University, Minia 61519, Egypt; 2 Department of Information Technology, College of Computer and Information Sciences, Princess Nourah bint Abdulrahman University, P.O. Box 84428, Riyadh 11671, Saudi Arabia

**Keywords:** search and rescue optimization algorithm, meta-heuristics, opposition-based learning, multi-level thresholding, fuzzy entropy and Otsu method, image segmentation, 68Txx, 68Uxx

## Abstract

Image segmentation has been one of the most active research areas in the last decade. The traditional multi-level thresholding techniques are effective for bi-level thresholding because of their resilience, simplicity, accuracy, and low convergence time, but these traditional techniques are not effective in determining the optimal multi-level thresholding for image segmentation. Therefore, an efficient version of the search and rescue optimization algorithm (SAR) based on opposition-based learning (OBL) is proposed in this paper to segment blood-cell images and solve problems of multi-level thresholding. The SAR algorithm is one of the most popular meta-heuristic algorithms (MHs) that mimics humans’ exploration behavior during search and rescue operations. The SAR algorithm, which utilizes the OBL technique to enhance the algorithm’s ability to jump out of the local optimum and enhance its search efficiency, is termed mSAR. A set of experiments is applied to evaluate the performance of mSAR, solve the problem of multi-level thresholding for image segmentation, and demonstrate the impact of combining the OBL technique with the original SAR for improving solution quality and accelerating convergence speed. The effectiveness of the proposed mSAR is evaluated against other competing algorithms, including the L’evy flight distribution (LFD), Harris hawks optimization (HHO), sine cosine algorithm (SCA), equilibrium optimizer (EO), gravitational search algorithm (GSA), arithmetic optimization algorithm (AOA), and the original SAR. Furthermore, a set of experiments for multi-level thresholding image segmentation is performed to prove the superiority of the proposed mSAR using fuzzy entropy and the Otsu method as two objective functions over a set of benchmark images with different numbers of thresholds based on a set of evaluation matrices. Finally, analysis of the experiments’ outcomes indicates that the mSAR algorithm is highly efficient in terms of the quality of the segmented image and feature conservation, compared with the other competing algorithms.

## 1. Introduction

Image thresholding is a popular operation used in computer vision to process and analyze images in fields such as medicine, engineering, agriculture, and manufacturing. It is most commonly used in image segmentation to provide accurate feature extraction in biological image processing, pattern recognition, and robotic vision [1]. Thresholding is one of the most important main segmentation steps that has proven effective in different applications [2,3]. The main goal of image thresholding is to obtain optimal threshold values from the image, for which the image histogram is used. The histogram is a vital step in the segmentation methods for defining the probability distribution value of pixels in the image [4]. The thresholding technique can be classified into two different groups: multi-level and bi-level thresholding. Multi-level thresholding usins two or more thresholding values to split an image into many different groups, while bi-level thresholding uses one threshold value to split an image into two groups [5,6].

Recently, many researchers have illustrated the capability of MHs to solve a diversity of complex optimization problems in different fields, such as biomedical [7], engineering [8,9], medical [10,11], communications [12], image segmentation [13], and feature selection [14]. MHs are considered flexible, non-derived, and highly clever in obtaining an optimal solution. Furthermore, these algorithms are considered the best method for providing the best solutions for complex optimization problems with the latest developments in computer techniques. Due to their advantages, MHs have become widely utilized to realize the optimal thresholds of color and gray-level images. MHs applied the search procedure to a problem landscape with a set of search agents that act as candidate solutions created repeatedly in an iterative procedure using heuristic operators. These operators, when utilized in various orders, generate different search strategies. In the same context, MHs are classified into four main groups: swarm-based algorithms, natural evolution-based algorithms, human-based algorithms, and physics-based algorithms [15,16].

As per published researches on swarm-based algorithms (SA), SA mimics the behavior of an organism within groups. Organisms usually interact with one another to achieve the best collective behavior [17]. Some works published in these offshoots, such as particle swarm optimization (PSO) [18], mimic the hunting behavior of birds and fish swarms. The natural evolution-based algorithms imitate biological evolution processes such as recombination, crossover, mutation, and feature inheritance in offspring [19]. The fitness function determines the quality of candidate solutions to the optimization problems, which work as individuals in a population. The genetic algorithm (GA) [20] and differential evolution (DE) [21] are two evolutionary algorithms inspired by biological evolution. The third category of MHs is human-based algorithms that mimic gregarious human attitudes. Many algorithms belong to these branches, such as the heap-based optimizer (HBO) [22], election campaign algorithm (ECA) [23], and teaching–learning-based optimization (TLBO) [24]. The fourth category of MHs is called physics-based algorithms, and these are inspired by physics to create factors that allow for the search of the best solution within the search scope. Many algorithms have been published in this branch, such as the electromagnetism-based algorithms (EMO) [25] and the GSA algorithm [26].

In the literature, several theories have been proposed to explain the efficacy of MHs on image thresholding [27,28]. There are several examples of MHs in this field; nevertheless, the following are a few notable state-of-the-art research efforts. In [29], the moth-swarm algorithm (MSA) was used to determine the optimal threshold values with the Kapur method. Additionally, ant colony optimization (ACO) in [30] is utilized in image segmentation based on a non-local 2D histogram and Kapur’s entropy as object functions with the multi-threshold image segmentation (MTH) method. In [31], researchers used the Otsu and Kapur methods with a modified firefly algorithm to process images. In the same context, three new versions of the manta ray foraging optimization algorithm and the chimp optimization algorithm (ChOA) have been proposed to tackle the image segmentation problem using multi-level thresholding [32,33,34]. The researchers in [35] used (MTH) image segmentation with a novel concept of MHs called hyper-heuristics, in which each iteration defined the best execution sequence of MHs to determine the best thresholds. In [27], the black widow optimization (BWO) algorithm used Otsu or Kapur techniques as an objective function with multi-level thresholding to determine the optimal thresholding value in the gray level. An efficient krill herd (EKH) algorithm in [36] was utilized to determine the best thresholding values at different levels for color images, with Kapur’s entropy, Tsallis entropy, and the Otsu methods. The HHO algorithm is the novel algorithm in [37], and the hybridization of HHO is accomplished by adding another efficient algorithm, the DE algorithm, which together is termed HHO-DE. In particular, the entire population is divided into two equal subpopulations that will be assigned to the DE and HHO algorithms, respectively, and this hybridization used Otsu and Kapur’s methods as the objective functions.

Regardless of the previous optimization methods, SAR [38] is also used to determine the optimal multi-level thresholding for image segmentation. In terms of efficiency and simplicity, the SAR outperforms several other biologically inspired procedures as a competitive and modern population-based optimization technique. It mimics the exploration behavior of humans during search and rescue operations. The researchers [38] have proven that SAR performs better in terms of stability and accuracy when compared with other optimization techniques when tested on standard benchmark functions.

Many of the MHs used to solve various optimization problems in the literature, such as a lack of global search capability, early conversion, or trapping in local regions. This gives researchers a yardstick to propose hybrid and modified versions. Many researchers use OBL to improve the search efficiency of MHs [39]. Tizhoosh released the first version of OBL in 2005 [40], and MHs have used it in a variety of ways to improve exploratory searchability. The researchers improved the elephant herding optimization (EHO) using dynamic Cauchy mutation (DCM) and OBL learning for solving multi-thresholding problems [41]. DCM healed premature convergence, and OBL resolved slow convergence in EHO, according to the authors. This study utilized Otsu and Kapur’s methods as two fitness functions to determine threshold values for image segmentation. In [42], the marine predator algorithm (MPA) is improved using OBL (MPA-OBL) for enhancing convergence and search efficiency and solving the IEEE CEC’2020 benchmark problems. MPA-OBL used Kapur’s and the Otsu methods as two fitness functions with a diversity of benchmark images at different thresholds.

In spite of some studies related to MHs and OBL to solving image thresholding problems, it is difficult to find more research related to relevant literature. Nevertheless, OBL-based MHs are often applied to different optimization problems. As a result, the purpose of this study is to deepen the research in the image segmentation field by employing the most recent SAR; in reality, this is the first instance of SAR implemented on the image segmentation and CEC’2020 benchmark functions. SAR was introduced in July 2020 to mimic the exploration behavior of humans during search and rescue operations, which outperformed a huge number of well-known counterparts on a variety of engineering and mathematics benchmark problems. Despite the efficacy of SAR’s search mechanisms, there are some fields where it may be enhanced further to prove its efficiency on various optimization problems, such as image thresholding problems, because the algorithm may miss some critical search regions. To solve this problem, we combined the SAR algorithm with OBL (SAR-OBL) to generate solutions from potential regions for exploring search space more rigorously. More crucially, we enhance its local search capability by utilizing solutions generated from the surrounding promising regions to avoid the trap of local optima. As a result, the proposed SAR diversity comes with a trade-off balance between exploration and exploitation. We evaluate the proposed SAR-OBL on CEC’2020 benchmark functions before it is used to solve multi-level image thresholding problems.

The main goal of this paper is to enable researchers to use a novel metaheuristic algorithm with opposition-based learning (OBL) to increase the performance of image segmentation in the medical field. Furthermore, this paper aims to discuss the benefits and downsides of the mSAR algorithm in image segmentation. Finally, the main contributions of this paper can be summarized as follows:

1. An improved opposition-based search and rescue optimization algorithm (mSAR) is presented.
2. The search efficiency and convergence of mSAR have been improved.
3. Fuzzy entropy and the Otsu method are applied.

The paper is structured as follows: in Section 2, the SAR algorithm and OBL search strategy will be discussed. Section 2.3 is devoted to a mathematical model of fuzzy entropy and Otsu methods. Section 3 presents the proposed algorithm. The evaluation of SAR-OBL is presented in Section 4. The experimental results are discussed and analyzed in Section 5. The conclusion is reported in Section 6.

## 2. Preliminaries

This section studies the inspiration of the SAR algorithm and its mathematical model and discusses the OBL strategy.

### 2.1. Search and Rescue Optimization Algorithm

The mathematical model of the SAR algorithm is discussed in this section. Search and rescue operations are one type of group exploration. A search is a methodical operation that employs resources and available personnel to identify the individuals involved in the ordeal. A rescue operation seeks to recover people who are in danger and transport them to a safe location. Humans search in groups, and each searching group regulates its practice to be ready for its respective operations. Based on their training, humans can find clues and traces of missing people. The found clues are of varying value and contain varying amounts of information regarding the missing individuals. For example, some clues refer to the probability of the presence of lost individuals in that area. The clues are evaluated by each group member depending on his or her training. This evaluation delivers information about the found clues to other members via communication equipment. Last but not least, they search based on the information gleaned from them and the significance of these hints. Social and individual stages are the main stages in search and rescue operations. In the social stage, searching is based on the location of found clues and their importance, while in the individual stage, the search is executed regardless of the importance of clues. Clues can be divided into two types: hold clues and abandoned clues.

#### 2.1.1. The Clues

The positions of the left clues are saved in a memory matrix *X* in the model we proposed, while the positions of the humans are saved in a position matrix *Y*. The dimensions of matrix *X* are the same as those of matrix *Y*. They are M×N matrices, where *M* is the number of the members of the group and *N* refers to the problems’ dimension. The clues matrix *Z* contains the locations of the discovered clues, and it is composed of matrices *Y* and *X*. Most new solutions in individual and social stages are introduced dependent on the clue matrix, and this is a primary part of SAR. Equation (1) presents the clue matrix *Z* and matrices *X*, *Y*, and *Z* are upgraded in each of the human search stages.

(1) $$Z=\left[\begin{array}{c} Y\\ X \end{array}\right]=\left[\begin{array}{ccc} Y_{11} & \cdots & Y1N\\ \vdots & \ddots & \vdots \\ Y_{M1} & \cdots & Y_{MN}\\ X_{11} & \cdots & X_{1N}\\ \vdots & \ddots & \vdots \\ X_{M1} & \cdots & X_{MN} \end{array}\right],$$

where *Y* and *X* are human locations and memory matrices, respectively, and $Y_{M1}$ is the first dimension location for human *M*th. Furthermore, the first memory’s *N*th dimension is located at $X_{1N}$. The two stages of human search are modeled in the following section.

#### 2.1.2. Social and Individual Stages

According to the preceding explications, Equation (2) is utilized to determine the search direction.

(2) $$SD_{k}=\left(Y_{k}-Z_{j}\right),j\neq k$$

where the location of the *j*th clue, the location of the *k*th human, and the search direction for the *k*th human are $Z_{j}$, $Y_{k}$, and $SD_{k}$, respectively. *j* is a random parameter inside (1, 2M). For $k=j,Z_{k}$ will be equal to $Y_{k}$. Thus, *j* is elected in such a way that j≠k. The social phase can be implemented by Equation (3).

(3) $$Y_{j,i}^{\prime }=\left\{\begin{array}{cc} \left\{\begin{array}{cc} Z_{k,i}+r1\times \left(X_{j,i}-Z_{k,i}\right) & \;\mathrm{if}\;f\left(Z_{k}\right)>f\left(X_{j}\right)\\ X_{j,i}+r1\times \left(X_{j,i}-Z_{k,i}\right) & \;\mathrm{otherwise}\; \end{array}\right.\\ X_{j,i} & \;\mathrm{if}\;r2<SE\;\mathrm{or}\;i=i_{\mathrm{rand}\;}i=1,\cdots ,N \end{array}\right.,$$

where the location of the *i*th dimension for the *j*th human is $Y_{j,i}^{\prime }$. The new location of the *i*th dimension for the *k*th clue is $Z_{k,i}$. The fitness function values for the solution $X_{j}$ and $Z_{k}$ are $f\left(X_{j}\right)$ and $f\left(Z_{k}\right)$, respectively. r1 is a random parameter inside [−1,1], r2 and SE are a distributed random parameters inside range [0,1]. $i_{\mathrm{rand}\;}$ is a random parameter inside range 1 and *N* which used to ensure that at least one dimension of $Y_{j,i}^{\prime }$ is different from $X_{j,i}$. Equation (3) shows the new location of the *j*th human in all dimensions.

The individual stage used Equation (4) to determine the new location of the *j*th human.

(4) $$Y_{j}^{\prime }=X_{j}+r3\times \left(C_{k}-C_{m}\right),j\neq k\neq m.$$

where *k*, *m* are random values ranging between 1 and 2N. This way, j≠k≠m was employed for choosing *k* and *m* to prevent moving along other clues. r3 is a random value with a uniform distribution inside the range [0,1]. Equation (5) is used to modify the new location of the *j*th human.

(5) $$Y_{j,i}^{\prime }=\left\{\begin{array}{cc} \left(X_{j,i}+X_{i}^{\max}\right)/2 & \;\mathrm{if}\;X_{j,i}^{\prime }>X_{i}^{\max}\\ \left(X_{j,i}+X_{i}^{\min}\right)/2 & \;\mathrm{if}\;X_{j,i}^{\prime }<X_{i}^{\min} \end{array},i=1,\cdots ,N\right.,$$

where $X_{i}^{\min\;}$, $X_{i}^{\max}$ are the values of the minimum and maximum threshold for *i*th dimension, respectively.

#### 2.1.3. Update Locations and Information

Members of the group will search according to these two stages in each iteration, and if the value of the fitness function after each stage in the first location is $\left(f\left(X_{j}\right)\right)$ less than the second location $Y_{j}^{\prime }\left(f\left(Y_{j}^{\prime }\right)\right)$. The first location $\left(X_{j}\right)$ will be saved in a random location of the memory matrix (*M*).

(6) $$M_{n}=\left\{\begin{array}{cc} X_{j} & \;\mathrm{if}\;f\left(Y_{j}^{\prime }\right)>f\left(X_{j}\right)\\ M_{n} & \;\mathrm{otherwise}\; \end{array}\right.,$$

(7) $$X_{j}=\left\{\begin{array}{cc} Y_{j}^{\prime } & \;\mathrm{if}\;f\left(Y_{j}^{\prime }\right)>f\left(X_{j}\right)\\ X_{j} & \;\mathrm{otherwise}\; \end{array}\right.,$$

where $M_{n}$ indicates the location of the *n*th saved clue in the memory matrix and the random value ranged between 1 and *N* is *n*.

#### 2.1.4. Constraint-Handling Strategies

The penalty functions strategy, the ε-constrained method, and stochastic ordering are all methods for dealing with constraints. The ε-constrained method is one of the prevalent constraint-handling methods used in this study. According to this strategy, for a maximizing problem, this technique considers one solution to be better than another solution if the following requirements are satisfied:

(8) $$X_{2}\;\mathrm{is}\;\mathrm{better}\;\mathrm{than}\;X_{1}:\left\{\begin{array}{cc} f\left(X_{2}\right)>f\left(X_{1}\right) & \;\mathrm{if}\;G\left(X_{2}\right)\leq \varepsilon \;\mathrm{and}\;G\left(X_{1}\right)\leq \varepsilon \\ f\left(X_{2}\right)>f\left(X_{1}\right) & \;\mathrm{if}\;G\left(X_{2}\right)=G\left(X_{1}\right)\\ G\left(X_{2}\right)<G\left(X_{1}\right) & \;\mathrm{otherwise}\; \end{array}\right..$$

The parameter ε is used to control the size of feasible space and this parameter can be calculated by Equation (8).

The SAR algorithm is compared using the ε-constrained technique for constraint optimization problems. Thus, Equations (3), (6) and (7) are updated as follows:

(9) $$Y_{j,i}^{\prime }=\left\{\begin{array}{cc} \left\{\begin{array}{cc} Z_{k,i}+r1\times \left(X_{j,i}-Z_{k,i}\right) & \;\mathrm{if}\;f\left(Z_{k}\right)\;\mathrm{is}\;\mathrm{better}\;\mathrm{than}\;f\left(X_{j}\right)\\ X_{j,i}+r1\times \left(X_{j,i}-Z_{k,i}\right) & \;\mathrm{otherwise}\; \end{array}\right.\\ X_{j,i} & \;\mathrm{if}\;r2<SE\;\mathrm{or}\;i=i_{\mathrm{rand}\;}i=1,\cdots ,N \end{array}\right.,$$

(10) $$M_{n}=\left\{\begin{array}{cc} Y_{j}^{\prime } & \;\mathrm{if}\;Y_{j}^{\prime }\;\mathrm{is}\;\mathrm{better}\;\mathrm{than}\;X_{j}\\ M_{n} & \;\mathrm{otherwise}\; \end{array}\right.,$$

(11) $$X_{j}=\left\{\begin{array}{cc} Y_{j}^{\prime } & \;\mathrm{if}\;Y_{j}^{\prime }\;\mathrm{is}\;\mathrm{better}\;\mathrm{than}\;X_{j}\\ X_{j} & \;\mathrm{otherwise}\; \end{array}\right.,$$

where $Z_{k}$ or $Y_{j}^{\prime }$ are better than $X_{j}$ if and only if Equation (8) is satisfied.

### 2.2. Opposition-Based Learning

OBL is a new mechanism in computational intelligence, and the main principle of OBL is to enhance the efficiency of the optimization OBL of MHs [42]. HR. Tizhoosh [40] introduced the OBL. Many researchers use OBL to enhance the quality of their population-initiated solutions by diversifying these solutions. In general, in MHs, when the initial solutions are near the optimal location, convergence occurs quickly; moreover, late convergence is expected. In this case, the OBL technique provides additional solutions by taking into account opposite search regions that may be near the global optimum. For understanding the definition of OBL, the opposite point of *P* is computed by:

(12) $${\vec{z}}_{j}=\left({\vec{ub}}_{j}+{\vec{lb}}_{j}\right)-{\vec{z}}_{j},$$

where ub and lb are upper and lower bounds for the *j*th component of a vector *z*.

Previous studies indicate that researchers in the MH community have predominantly used various MHs. For instance, in [43], the HHO algorithm used the OBL strategy to enhance the search efficiency of the algorithm. While the researchers in [44] presented a new version of SCA based on OBL, this version is designed to jump out of local optima during the search processes. An improved whale optimization algorithm (WOA) is developed in [45] by combining the OBL and DE algorithms for improving exploration and exploitation by generating opposite values. In [46], researchers used OBL to improve the grasshopper optimization algorithm to solve engineering problems and benchmark functions. The EO algorithm used OBL and a set of novel update rules in [47] for modifying the EO, and this modification is called m-EO. The shuffled frog-leaping algorithm (SFLA) used the OBL to improve SFLA in [48], where OBL is combined into the memeplexes before the frog initiates foraging. In [49], researchers combine OBL with an enhanced scatter search (eSS) for large-scale parameter estimation in kinetic models of medical fields. According to this research work, OBL is employed only during the initiation phase for improving the rate of convergence and avoiding tumbling into the local optima of SAR. After that, mSAR is utilized for solving the problem of multi-thresholding for image segmentation using fuzzy entropy and the Otsu variance as objective functions.

### 2.3. Image Thresholding Methods

In this subsection, the mathematical model of two object functions used in image thresholding is explained, and these object functions are fuzzy entropy [50] and Otsu variance [51].

#### 2.3.1. Fuzzy Entropy

In fuzzy entropy [52], the size of an image is termed as N∗M, and the original image *I* will be D={(j,i)∣j=0,…,N−1;i=0,…,M−1}. $T_{1}$ and $T_{2}$ are two thresholds for dividing the image into three regions $E_{u},E_{m},E_{l}$, in which these regions define the pixels of high gray levels, pixels of low gray levels, and pixels of middle gray levels repetitively. Usually, Equation (13) is utilized to calculate the histogram of the image as follows:

(13) $$H_{k}=\frac{n_{k}}{N*M},\;\sum_{k=0}^{NM}H_{k}=1,$$

where *k* is an intensity level inside ranged (0≤k≤L−1). $n_{k}$ is the number of occurrences of the *k* in the image represented by the histogram. The probability distribution of gray level *k* is $H_{k}$. The probability distribution of $E_{u}$, $E_{m}$, and $E_{l}$ can be computed as:

(14) $$p_{u}=P\left(E_{u}\right);\;p_{m}=P\left(E_{m}\right);\;p_{l}=P\left(E_{l}\right),$$

**Figure 1:**
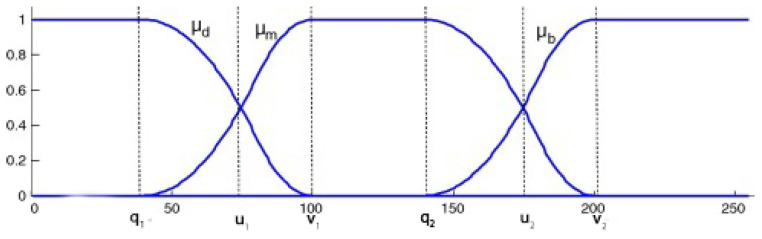
Membership function graph.

We choose $\mu_{d},\mu_{m},\mu_{b}$ as the membership functions of $E_{u}$, $E_{m}$, $E_{l}$ as shown in Figure 1. There are six fuzzy parameters, and these are $q_{1}$, $u_{1}$, $v_{1}$, $q_{2}$, $u_{2}$, $v_{2}$. Therefore, these parameters are used to determine $T_{1}$ and $T_{2}$. According to the previous statements, the probability distribution of three regions can be calculated by the following formula:

(15) $$\begin{array}{cc} p_{u} & =\sum_{k=0}^{255}p_{k}*p_{u|k}=\sum_{k=0}^{255}p_{k}*\mu_{d}\left(k\right),\\ p_{m} & =\sum_{k=0}^{255}p_{k}*p_{m|k}=\sum_{k=0}^{255}p_{k}*\mu_{m}\left(k\right),\\ p_{l} & =\sum_{k=0}^{255}p_{k}*p_{l|k}=\sum_{k=0}^{255}p_{k}*\mu_{b}\left(k\right), \end{array}$$

where the conditional probability of a pixel that is classified into three partitions are $p_{u/k},p_{m/k},p_{l/k}$ and satisfy the constraint of $p_{u/k}+p_{m/k}+p_{l/k}=1$.

The mathematical model of the three membership functions is defined as follows:

(16) $$\mu_{d}\left(k\right)=\left\{\begin{array}{cc} 1 & k\leq q_{1}\\ 1-\frac{{\left(k-q_{1}\right)}^{2}}{\left(v_{1}-q_{1}\right)*\left(u_{1}-q_{1}\right)} & q_{1}\leq k\leq u_{1}\\ \frac{{\left(k-v_{1}\right)}^{2}}{\left(v_{1}-q_{1}\right)*\left(v_{1}-u_{1}\right)} & u_{1}\leq k\leq v_{1}\\ 0 & k\geq v_{1} \end{array}\right.,$$

(17) $$\mu_{m}\left(k\right)=\left\{\begin{array}{cc} 0 & k\leq q_{1}\\ \frac{{\left(k-q_{1}\right)}^{2}}{\left(v_{1}-q_{1}\right)*\left(u_{1}-q_{1}\right)} & q_{1}\leq k\leq u_{1}\\ 1-\frac{{\left(k-v_{1}\right)}^{2}}{\left(v_{1}-q_{1}\right)*\left(v_{1}-u_{1}\right)} & u_{1}\leq k\leq v_{1}\\ 1 & v_{1}\leq k\leq q_{2} \end{array}\right.,$$

(18) $$\mu_{b}\left(k\right)=\left\{\begin{array}{cc} 0 & k\leq q_{2}\\ \frac{{\left(k-q_{2}\right)}^{2}}{\left(v_{2}-q_{2}\right)*\left(u_{2}-q_{2}\right)} & q_{2}\leq k\leq u_{2}\\ 1-\frac{{\left(k-v_{2}\right)}^{2}}{\left(v_{2}-q_{2}\right)*\left(v_{21}-u_{2}\right)} & u_{2}\leq k\leq v_{2}\\ 1 & k\geq v_{2} \end{array}\right.,$$

the six parameters $q_{1}$, $u_{1}$, $v_{1}$, $q_{2}$, $u_{2}$, $v_{2}$ must fulfill the condition $0\leq q_{1}<u_{1}<v_{1}<q_{2}<u_{2}<v_{2}\leq 255$. In order to reach the maximum value of entropy, a combination of the membership function’s variables must be found. As a result, the most applicable threshold can be computed as follows:

(19) $$\begin{array}{c} \mu_{d}\left(T_{1}\right)=\mu_{m}\left(T_{1}\right)=0.5,\\ \mu_{m}\left(T_{2}\right)=\mu_{b}\left(T_{2}\right)=0.5 \end{array}$$

According to the previous equations, the thresholds $T_{1}$ and $T_{2}$ can be computed by Equations (16)–(18) and the result is as following:

(20) $$\begin{array}{cc} T_{1} & =\left\{\begin{array}{cc} q_{1}+\sqrt{\left(v_{1}-q_{1}\right)*\left(u_{1}-q_{1}\right)/2} & \left(q_{1}+v_{1}\right)/2⩽u_{1}⩽v_{1}\\ v_{1}-\sqrt{\left(v_{1}-q_{1}\right)*\left(v_{1}-u_{1}\right)/2} & q_{1}⩽u_{1}⩽\left(q_{1}+v_{1}\right)/2 \end{array}\right.,\\ T_{2} & =\left\{\begin{array}{cc} q_{2}+\sqrt{\left(v_{2}-q_{2}\right)*\left(u_{2}-q_{2}\right)/2} & \left(q_{2}+v_{2}\right)/2⩽u_{2}⩽v_{2}\\ v_{2}-\sqrt{\left(v_{1}-q_{2}\right)*\left(v_{2}-u_{2}\right)/2} & q_{2}⩽u_{2}⩽\left(q_{2}+v_{2}\right)/2 \end{array}\right.. \end{array}$$

#### 2.3.2. Otsu Method

The Otsu technique [51] is one of the most commonly utilized segmentation methods for finding optimal threshold values by maximizing the class variance. The intensity levels *L* for each color image are within the range 0,1,2,…L−1. The probability distribution of the intensity value is computed as follows:

(21) $$Ph_{j}=\frac{N_{j}}{nk},Ph_{j}\geq 0,\sum_{j=1}^{L}Ph_{j}=1,$$

where nk denotes the number of pixels in the image and *j* is an intensity level in the range (0≤j≤L−1). The number of gray level *i* in the image represented by the histogram is $N_{i}$, and the probability distribution of the intensity levels is $Ph_{j}$. For bi-level segmentation, the classes are computed as follows:

(22) $$C_{a}=\frac{Ph_{1}}{\omega_{1}\left(th\right)},\ldots ,\frac{Ph_{th}}{\omega_{1}\left(th\right)}\;\mathrm{and}\;C_{b}=\frac{Ph_{th+1}^{c}}{\omega_{2}\left(th\right)},\ldots ,\frac{Ph_{L}}{\omega_{2}\left(th\right)}.$$

where $\omega_{1}\left(th\right)$ and $\omega_{2}\left(th\right)$ are the cumulative probability distributions for $C_{a}$ and $C_{b}$, respectively. $\omega_{1}\left(th\right)$ and $\omega_{2}\left(th\right)$ can be computed by the following:

(23) $$\omega_{1}\left(th\right)=\sum_{j=1}^{th}Ph_{j}\;\mathrm{and}\;\omega_{2}\left(th\right)=\sum_{th+1}^{L}Ph_{j}.$$

The mean levels $\mu_{1}$ and $\mu_{2}$ must be computed that define the classes using Equation (24). Equation (25) is used for computing the Otsu-based between-class $\sigma_{A}^{2}$.

(24) $$\mu_{1}=\sum_{j=1}^{th}\frac{jPh_{j}}{\omega_{1}\left(th\right)}\;\mathrm{and}\;\mu_{2}=\sum_{j=th+1}^{L}\frac{jPh_{j}}{\omega_{2}\left(th\right)},$$

(25) $$\sigma_{A}^{2}=\sigma_{a}+\sigma_{b}.$$

Additionally, $\sigma_{a}$ and $\sigma_{b}$ are the variance of $C_{a}$ and $C_{b}$ and are calculated as follows:

(26) $$\sigma_{a}=\omega_{1}{\left(\mu_{1}+\mu_{T}\right)}^{2}\;\mathrm{and}\;\sigma_{b}=\omega_{2}{\left(\mu_{2}+\mu_{T}\right)}^{2},$$

where $\mu_{T}=\omega_{1}\mu_{1}+\omega_{2}\mu_{2}$ and $\omega_{1}+\omega_{2}=1$ based on the values $\sigma_{a}$ and $\sigma_{b}$, the objective function is defined by Equation (27). As a result, the optimization problem is decreased to determine the intensity level that maximizes Equation (27):

(27) $$F_{otsu}\left(th\right)=\max\left(\sigma_{A}^{2}\left(th\right)\right)\;\mathrm{where}\;0\leq th\leq L-1,$$

where the Otsu method variance for a given th value is $\sigma_{A}^{2}\left(th\right)$. EBO methods are utilized for determining the intensity level th to maximize the objective function according to Equation (27). The objective or fitness function $F_{otsu}\left(th\right)$ can be modulated for multiple thresholds as:

(28) $$F_{otsu}\left(TH\right)=\mathrm{Max}\left(\sigma_{A}^{2}\left(th\right)\right)\;\mathrm{where}\;0\leq th\leq L-1\;\mathrm{and}\;j=\left[1,2,3,\ldots ,n\right],$$

where $TH=[th_{1},th_{2},\ldots ,th_{n}-1]$ illustrates a vector including MTH, and the variance is calculated by Equation (29).

(29) $$N\sigma_{A}^{2}=\sum_{j=1}^{n}\sigma_{j}=\sum_{j=1}^{n}\omega_{2}{\left(\mu_{2}-\mu_{T}\right)}^{2}.$$

$\mu_{j}$ is the mean of a class, $\omega_{j}$ is the occurrence probability, and *j* represents a specific class.

## 3. The Proposed mSAR

In this section, a new technique that improves SAR is explained in detail and called mSAR. The SAR algorithm is improved based on OBL as a local research strategy to reinforce the convergence of the algorithm by enhancing the variety of its solutions and avoiding the drawbacks of the random population. As a result, mSAR utilizes OBL in the initialization phase to improve the search process as follows:

(30) $$O_{j}=LB_{i}+UB_{i}-X_{j},j\in 1,2,\ldots ,n,$$

where $O_{j}$ is a vector-maintaining solution obtained by using OBL. $LB_{i}$ and $UB_{i}$ are the lower and upper bounds of the $i^{th}$ component of a vector *X*. Finally, the phases below are given further detail on the phases of the proposed image thresholding model.

### 3.1. Initialization Phase in mSAR

In the first phase, the proposed model begins by reading the image from the benchmark dataset, calculating the histogram of the selected image, and calculating the probability distribution of the selected images by Equation (13). In the next step, the algorithm initializes the mSAR parameters, such as problem dimensions *D*, population size *N*, boundaries of the search space ($LB_{i}$, $UB_{i}$), and the maximum iteration number *T*. The SAR, like many other optimization algorithms, begins by randomly initializing the first population $x^{0}$ and then storing the results. Following that, the OBL concept is used to compute the $O_{j}$ vector maintaining solution using Equation (30).

### 3.2. Optimization Phase

**Figure 2:**
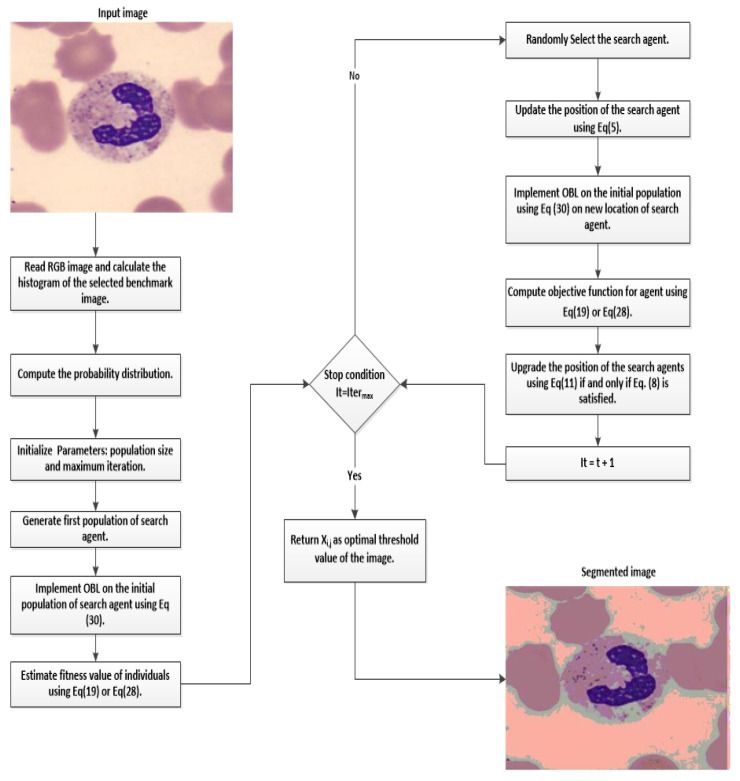
The flowchart of mSAR algorithm.

During this phase, we estimate the fitness values of the $X_{j}$ and $O_{j}$ populations to determine the optimal algorithmic threshold values. For updating the search agents’ locations, use the fitness value of the best threshold of the fuzzy entropy [52] or the Otsu [51] methods as the fitness function, and then compare the fitness values of $O_{j}$ and $X_{j}$ and store the optimal solution with the highest fitness. The optimization algorithm processes are split into two stages: the social and individual stages, as studied in the subsection on SAR. Following the implementation of the two phases of the optimization algorithm, each search agent updates its location repeatedly based on the best fitness values of $O_{j}$ and $X_{j}$, using the fuzzy entropy Equation (19) or the Otsu Equation (28), as described in Algorithm 1. The flowchart of the proposed mSAR algorithm is represented in Figure 2.

### 3.3. Final Phase of mSAR

After executing the optimization phase, fitness upgrade, memory storing, and search agents’ location upgrade using Equation (30), the proposed algorithm selects the optimal threshold values to generate the segmented image. The pseudo-code of the proposed algorithm is displayed in Algorithm 1.
**Algorithm 1** The proposed mSAR algorithm **Input**: Set parameters values (N=50, P=0.5, $T_{\max}=350$, t=30, SE=0.05); **Output**: Return the optimal solution with the optimal threshold values onto the image. Generate the first population $X_{j}^{0}$ randomly with dimensions *D*. Utilize Equation (30) to apply OBL on the first population $X_{j}^{0}$ and save outcomes in $O_{j}$. **while** $\left(t\leq Iter_{\max}\right)$ **do**  **for** j≤N **do**   Estimate $X_{j}$ utilizing fuzzy entropy Equation (19) or Otsu Equation (28) and save outcomes in $fit_{j}$.   Estimate $O_{j}$ utilizing fuzzy entropy Equation (19) or Otsu Equation (28) and save outcomes in $FitO_{j}$.   **if** $Fit_{j}<FitO_{j}$ **then**    $X_{j}=O_{j};$   **end if**  **end for**  Complete the memory saving.  **if** $t<\frac{Iter_{\max}}{3}$ **then**   Upgrade nominee solutions by Equation (9).  **else if** $\frac{Iter_{\max}}{3}<t<2\times \frac{Iter_{\max}}{3}$ **then**   Upgrade search agents’ location by Equation (11).  **else if** $t>2\times \frac{Iter_{\max}}{3}$ **then**   Upgrade nominee solutions by Equation (30).  **end if**  The optimal solution found so far will update.  **for** each candidate solution **do**   Estimate $X_{j}$ after upgrade by fuzzy entropy Equation (19) or Otsu Equation (28) and save outcomes in $fit_{j}$.   Estimate $O_{j}$ after upgrade by fuzzy entropy Equation (19) or Otsu Equation (28) and save outcomes in $FitO_{j}$.   **if** $Fit_{j}<FitO_{j}$ **then**    Set $X_{j}=O_{j};$   **end if**  **end for**  Execute memory storing and fitness updates.  Define the optimal value of search agents’ location.  Save the outcomes. **end while** Return the optimal solution with the optimal threshold values onto the image.

### 3.4. Computational Complexity of mSAR

This subsection illustrates the computational complexity of the proposed mSAR. Firstly, the complexity of the initialization process can be described as O(2×N×D), where *N* and *D* indicate the number of search agents and the dimension of the problem, respectively. During the initialization phase of the mSAR algorithm, sorting the search agent takes O(2Nlog(2N)) times. In the worst cases, the complexity of the social and individual phases is O(N×D). However, the complexity of the abandoning clue and restart processes in the worst cases are O(N×D). Furthermore, O(T) time complexity is required for mSAR to execute *T* of its main operations (initialization phase, social phase, abandoning clue, individual phase, restart strategy, and OBL). As a result, the mSAR computes the fitness of each agent with complexity as follows:

(31) $$\begin{array}{c} O(mSAR)=O\left(2\times N\times D\right)+O\left(2N\log\left(2N\right)\right)+t_{\max}\times (O\left(N\times D\right)\\ +O\left(N\times D\right)+O\left(N\times D\right)+O\left(N\times D\right)) \end{array}$$

Overall, the total computational complexity of the proposed mSAR can be represented by $O(T\times t_{\max}\times N\times D)$.

## 4. Performance Evaluation of mSAR on CEC’20 Test Suit

### 4.1. Parameter Settings of CEC’2020

**Figure 3:**
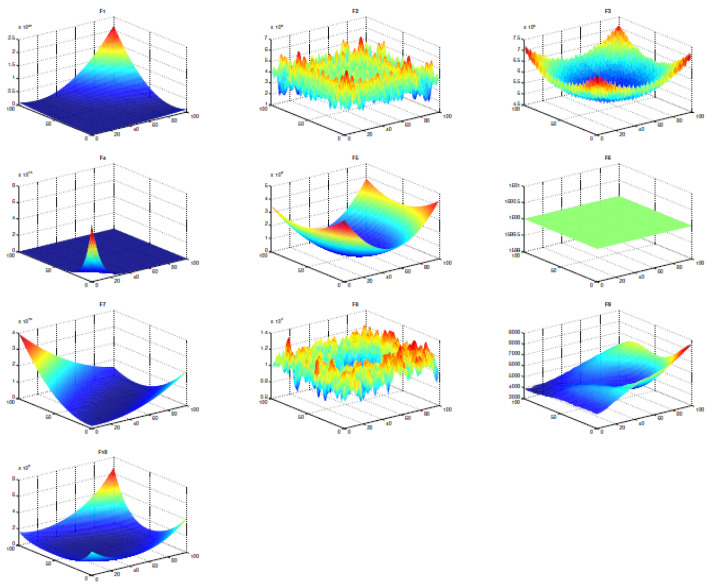
CEC’2020 benchmark functions in two-dimensional view.

In this subsection, the evaluation of the proposed mSAR algorithm is introduced. Therefore, the proposed mSAR algorithm used the IEEE Congress on Evolutionary Computation (CEC) [53] as a test problem for estimating the algorithms’ performance. The performance of mSAR is estimated over the CEC’2020 benchmark functions. Firstly, this benchmark function includes 10 test problems and can be classified into four types. This benchmark function includes unimodal, multimodal, hybrid, and composition functions. The mathematical formulation and parameter-setting of the CEC’2020 benchmark test are shown in Table 1; ’Fi*’ indicates the optimal global value. A two-dimensional visualization of the CEC’2020 functions is shown in Figure 3 to explain the differences and the nature of each problem.

### 4.2. Statistical Results Analysis

The performance of the proposed mSAR is estimated by utilizing the CEC’2020 benchmark test, which contains qualitative and quantitative metrics. The mean and standard deviation (STD) of optimal solutions obtained by the proposed algorithm and all other algorithms compared are quantitative metrics. Furthermore, the average fitness history, search history, and convergence curve are qualitative metrics. We utilized these qualitative metrics for estimating the performance of the proposed mSAR on the CEC’2020 benchmark test against seven well-known metaheuristic algorithms, namely LFD, HHO, SCA, EO, GSA, AOA, and the original SAR. The STD and mean of the optimum value produced from the proposed mSAR algorithm against other competing algorithms are illustrated in Table 2, and the optimum value of the mean and STD are minimum values in outcomes. According to the STD and mean results in Table 2, the proposed mSAR algorithm outperforms other competing algorithms on the CEC’2020 benchmark functions.

### 4.3. Boxplot Analysis of mSAR Algorithm

**Figure 4:**
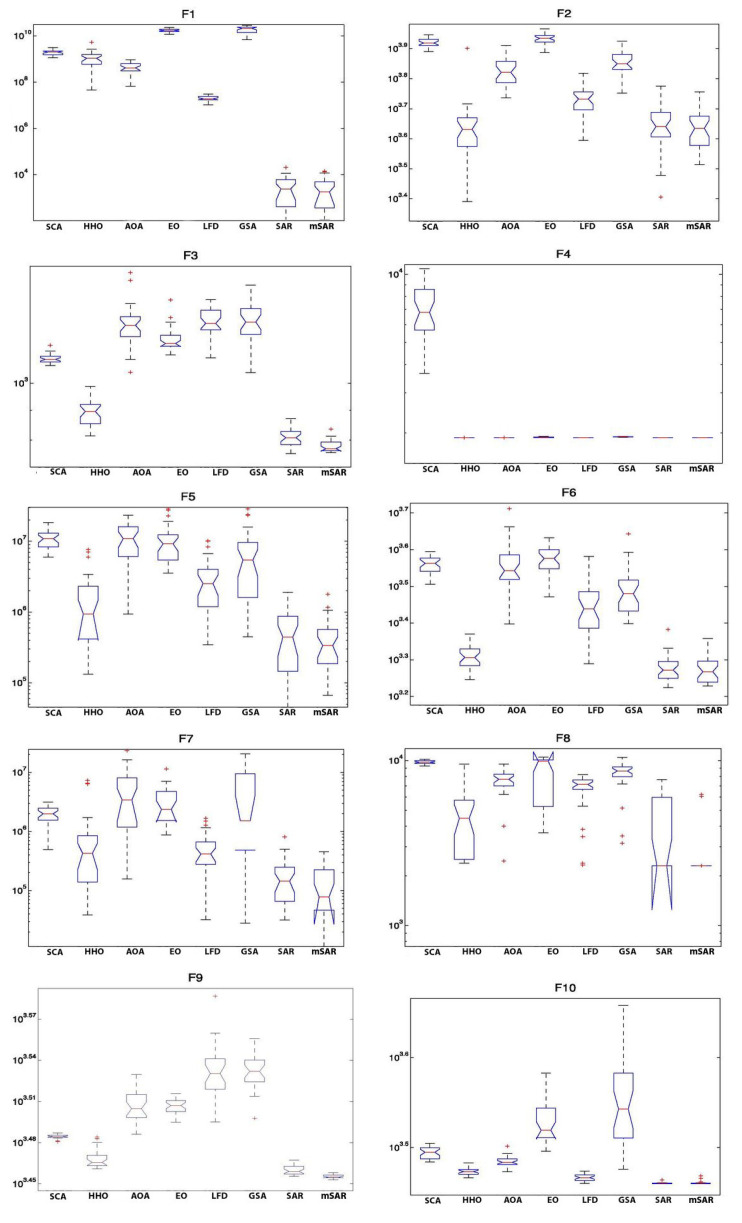
The outcomes of the boxplot of the proposed mSAR algorithm with other competitive algorithms over CEC’2020 functions with D=20.

Boxplot analysis is utilized to represent the data distribution characteristics. The boxplot analysis indicates the quality of the optimizers’ solutions and is used for reporting data with a homogeneous variance and a normal distribution. Figure 4 illustrates the outcomes of the boxplot of the proposed mSAR algorithm compared with other competitive algorithms. The boxplot is an excellent plot for showing data distributions in quartiles. The maximum and minimum are the highest and lowest data points acquired by the algorithm, which are the whiskers’ edges. These quartiles are the lower and upper quartiles, which are determined by the ends of the rectangles, and a narrow boxplot indicates the highest agreement among data. The outcomes of the boxplot of the proposed mSAR algorithm with other competitive algorithms and the outcomes of the ten functions boxplot for *D* = 20 are illustrated in Figure 4. Indeed, the proposed mSAR algorithm outperforms other competing algorithms on the majority of the benchmark functions, but its performance on F3 and F7 is limited.

### 4.4. Curves of Convergence of mSAR Algorithm

**Figure 5:**
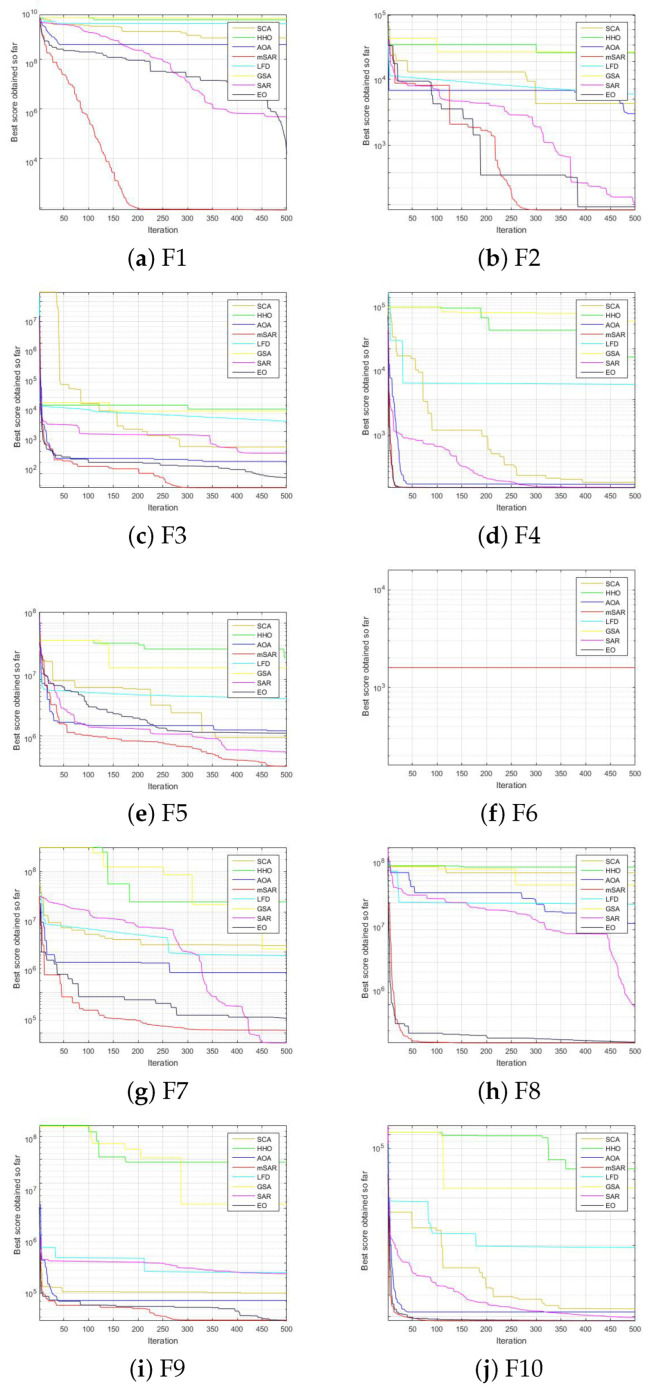
The convergence curves of the proposed mSAR algorithm with other competitive algorithms over CEC’2020 functions with D=20.

This subsection describes the convergence curves of the proposed SAR algorithm and other competing algorithms. The convergence curves of SCA, HHO, AOA, EO, LFD, GSA, SAR, and mSAR for the CEC’2020 functions are shown in Figure 5. Moreover, the mSAR algorithm reached a stable point for most functions and acquired optimal solutions for an algorithm. Therefore, most problems that need fast computation, such as online optimization problems, can be solved by the mSAR algorithm. The mSAR algorithm displayed stable behavior, and due to space limitations, its solutions converged quickly in most of the problems in which it was evaluated.

### 4.5. Qualitative Metrics

**Figure 6:**
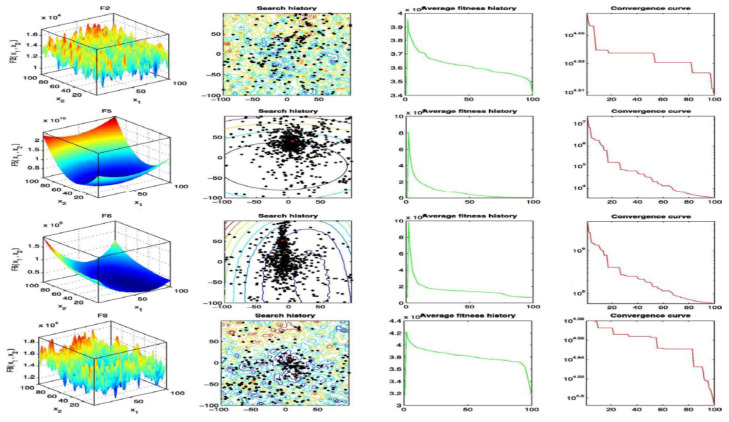
Qualitative metrics on F2, F5, F6, and F8: functions in 2D view, search history, average fitness history, and convergence curve.

Even though previous outcome studies assured the proposed mSAR algorithms’ superior performance, more experiments and analyses would help us draw more definitive conclusions regarding the algorithms’ performance in solving real problems. The qualitative analysis of the mSAR algorithm is illustrated in Figure 6. The first and second columns in the figure describe the CEC’2020 benchmark functions in two-dimensional space and show the search history of the agent. The average fitness history over 350 iterations is shown in the third column, which explains the agents’ overall behavior and their contribution to locating the optimum solution. Most of the history curves gradually decrease according to average fitness history, indicating that the population improves with each iteration. The optimization history and convergence curve are shown in the fourth column. The optimization history is reduced according to Figure 6. This means that the solution is optimized during iterations until the best solution is found.

## 5. Application of mSAR: Blood Cells Images Segmentation

The experimental environment of the proposed mSAR algorithm is illustrated in this section. The test images used for the experiments are presented first, followed by the empirical setup. Then, we analyzed the outcomes acquired by mSAR in terms of PSNR, SSIM, FSIM, and fitness.

### 5.1. Benchmark Images

**Figure 7:**
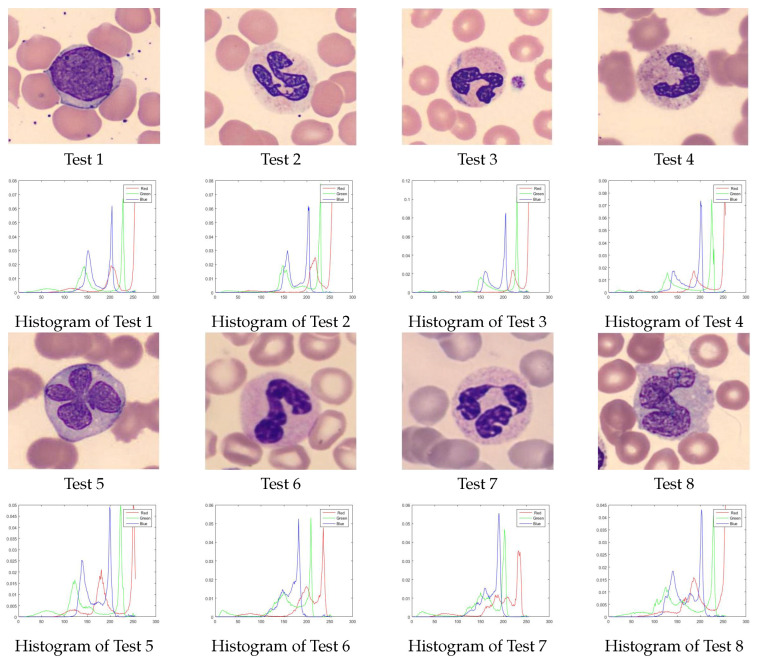
Set of test images and relative histograms.

To examine the performance of the proposed mSAR algorithm and determine the best threshold values, eight images of the benchmark were used. These images were taken in the core laboratory at the Hospital Clinic of Barcelona during the diagnosis of malaria infection [54]. The images in the dataset are in the format JPG (RGB, 2400 × 1800 pixels). These images have different features based on their histograms, as observed in Figure 7. The purpose of selecting these images is to test the performance and quality of the segmented images.

### 5.2. Experimental Setup

This subsection compares the proposed mSAR algorithm to seven metaheuristic algorithms: LFD [8], HHO [37], SCA [55], EO [56], GSA [26], AOA [57], and the original SAR [38]. All experiments were implemented in Matlab 2015 and executed on an Intel Core I5 with 6 GB of RAM running in a Windows 8.1 64-bit environment. To achieve a fair comparison, all competitor algorithms executed 30 runs per test image, the maximum number of iterations was set to 350, and the population was 50. Each algorithm’s parameter settings were defined using standard criteria and information from previous literature (default values). All experiments were executed on images using various thresholds: are $th_{2},th_{5},th_{7},$ and $th_{10}$ according to related literature [58]. Table 3 presents the parameter settings of the mSAR algorithm and its value.

### 5.3. Performance Metrics

Five metrics were used for evaluating the quality of the image and comparing the edges of the segmented images: Wilcoxon rank test, standard deviation (STD), peak signal-to-noise ratio (PSNR) [59], feature similarity (FSIM) [60], and structural similarity (SSIM) [61]. The algorithm becomes more stable when the value of STD is decreased by [62]. The STD can be defined as follows:

(32) $$STD=\sqrt{\sum_{j=1}^{n}\frac{\sigma_{j}-\mu }{n}},$$

where *n* is the total number of pixels in the image, μ is the mean of pixels, and $\sigma_{j}$ is the value of *j*th in the image.

#### 5.3.1. Peak Signal-To-Noise Ratio (PSNR)

PSNR [59,60] is a metric utilized for estimating the performance of the segmented image and computing the difference between the original image and its segmented image, and defined as follows:

(33) $$\begin{array}{cc} PSNR & =20\log_{10}\left(\frac{255}{RMSE}\right),\\ RMSE & =\sqrt{\left(\frac{\sum_{j=1}^{n_{r}}\sum_{i=1}^{n_{c}}\left({\left(I\left(j,i\right)-Seg\left(j,i\right)\right)}^{2}\right)}{n_{r}\times n_{c}}\right)}. \end{array}$$

In Equation (33), *I* is the original image, and Seg is the segmented image of size $n_{r}\times n_{c}$. The root-mean-squared error is RMSE.

#### 5.3.2. Feature Similarity Index (FSIM)

FSIM [61] is another measure used to map the features and measure the similarities based on the internal features of the image. FSIM is computed by the following:

(34) $$FSIM=\frac{\sum_{u\epsilon \Omega }S_{L}\left(u\right)PC_{n}\left(u\right)}{\sum_{u\epsilon \Omega }PC_{n}\left(u\right)},$$

where the entire domain of the image is Ω, and the value of $PC_{n}\left(u\right)$ is described as follows:

(35) $$PC=\frac{E(\omega )}{\left(\epsilon +\sum_{n}A_{n}\left(\omega \right)\right)},$$

where the magnitude of the vector in ω on *n* is E(ω), and the amplitude’s local on scale *n* is $A_{n}\left(\omega \right)$. ϵ is the small positive value and $PC_{n}\left(u\right)=\max\left(PC_{1}\left(u\right),PC2\left(u\right)\right)$.

#### 5.3.3. Structural Similarity Index (SSIM)

The value of SSIM is higher when the original image will be better segmented because the structures in the two images match. SSIM is defined in Equation (36).

(36) $$SSIM\left(I,Seg\right)=\frac{\left(2\mu_{1}\mu_{Seg}+c_{a}\right)\left(2\sigma_{1,Seg}+c_{b}\right)}{\left(\mu_{I}^{2}+\mu_{Seg}^{2}+c_{a}\right)\left(\sigma_{I}^{1}+\sigma_{Seg}^{2}+c_{b}\right)},$$

where the mean value of segmented image (Seg) and the original image *I* are $\mu_{Seg}$ and $\mu_{I}$, respectively. The standard deviation values of segmented image (Seg) and the original image *I* are $\sigma_{Seg}$ and $\sigma_{I}$, respectively. $c_{a}$ and $c_{b}$ are two constant numbers, and the covariance of *I* and Seg is $\sigma_{I,Seg}$.

#### 5.3.4. Wilcoxon Rank-Sum Test

The Wilcoxon rank-sum test is a non-parametric test that determines if two independent samples come from the same population distribution [63]; it is also utilized to compare the results obtained by algorithms. The test is constructed with two hypotheses in mind: the null hypothesis and the alternative hypothesis. According to the values of (P) and (H), the value of the null hypothesis is true when (*p* > 0.05) or (H=0), but the value of the alternative hypothesis is true when (*p* < 0.05) or (H=1).

#### 5.3.5. Friedman Mean Rank Test

The Friedman test is also a non-parametric statistical test utilized to find differences in treatments across three or more matched groups and evaluate the performance of the competitive algorithms. This statistical test is utilized for one-way repeated measures analysis of variance by the value of ranks and was generated by Milton Friedman [64]. The values of the mean ranked are computed by Friedman’s statistics [64]. Friedman’s statistics compare the critical values obtained from competitors’ algorithms to see whether the null hypothesis is rejected or not.

### 5.4. Results Analysis of the Proposed Algorithm Based on Fuzzy Entropy

This subsection discusses and analyzes the outcomes of the proposed algorithm mSAR using different numbers of thresholds [nTh=2,5,7,10]. The fuzzy entropy method is used in this section as an objective function to obtain optimal thresholds.

**Figure 8:**
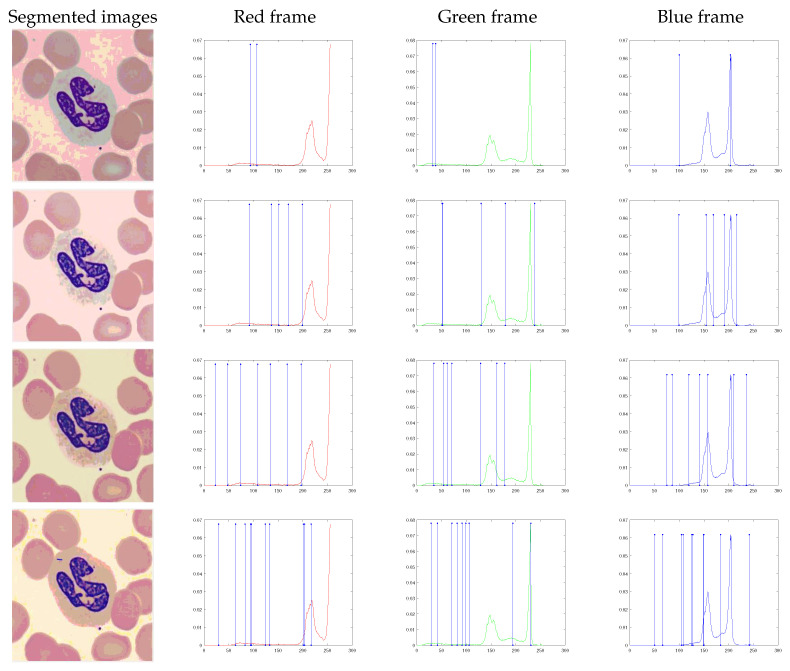
Threshold outcomes of each layer of a color image using the mSAR algorithm with the fuzzy entropy method.

**Figure A1:**
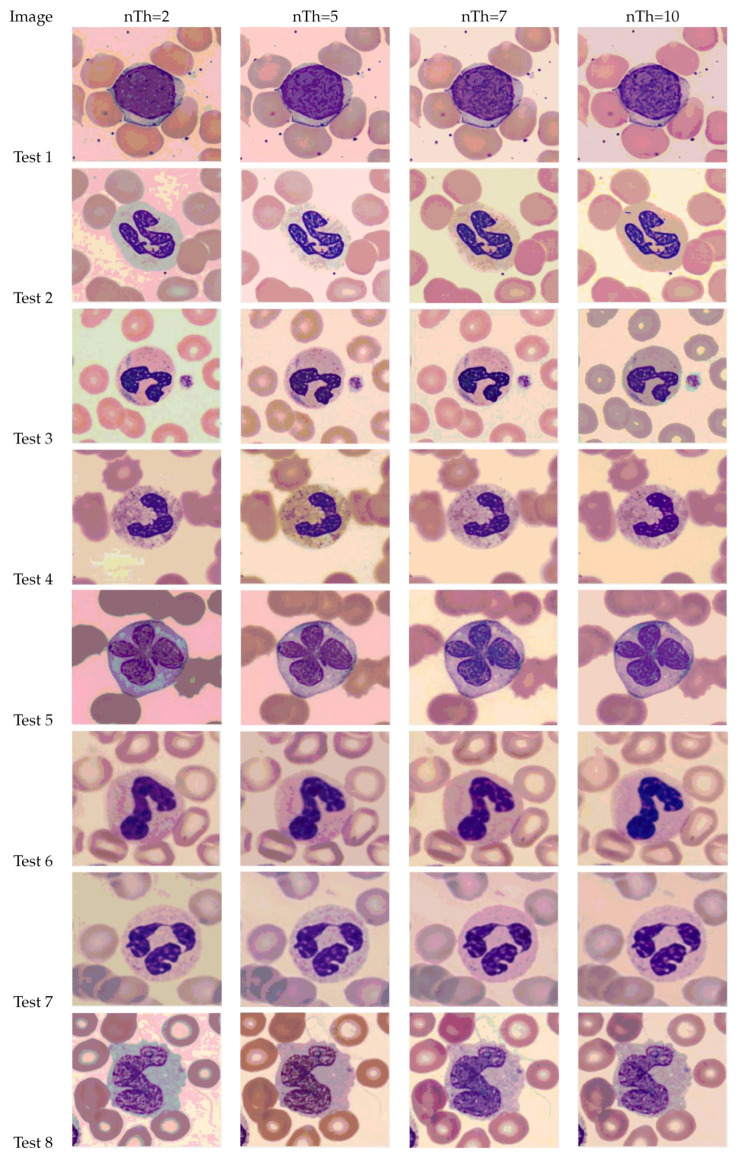
Outcomes after executing mSAR on Fuzzy Entropy’s method over the set of test images.

Figure A1 in Appendix A illustrates the resulting images of the mSAR algorithm with various numbers of thresholds for all the test images utilized in the experiments. Figure 8 illustrates the resulting images of the mSAR with a different number of thresholds over test image 2 and presents a graphical analysis of the thresholds of the segmented images. The optimal thresholding values obtained by the mSAR algorithm and the original SAR algorithm using the fuzzy entropy method are shown in Table A1 in Appendix A. The mean and STD outcomes of the fitness, PSNR, SSIM, and FSIM are illustrated in Table 4, Table 5, Table 6 and Table 7, respectively. When the mean value of an algorithm is high, the algorithm is efficient and accurate. Hwoever, when the value of the STD of an algorithm is low, the algorithm is more stable.

Table 4 displays the mean and STD of the fitness outcomes for the test images using fuzzy entropy as an objective function. According to these outcomes, the proposed mSAR algorithm is in the first position with 22 higher fitness values in 32 experiments, followed by the original SAR in the second position with six experiments, while the SCA and EO obtained the optimal fitness values in four experiments, and HHO comes in the fourth position with two experiments. GSA is in the fifth position with only one experiment, and the remaining algorithms, LFD and AOA, obtained zero higher fitness values. The values of STD computed for the 32 independent outcomes for all benchmark images with a different number of thresholds are shown in Table 4, and the algorithm is more stable when the value of STD is lower.

The mean and STD of the PSNR outcomes for all test images are illustrated in Table 5. The mSAR algorithm comes in the first position in terms of the mean values of PSNR with 20 experiments, while SCA is in the second position with six experiments. The original SAR and AOA come in the third position in only four experiments, followed by HHO in the fourth position in three experiments. Finally, the worst result was obtained by EO, which obtained only one experiment, while GSA and LFD could not obtain the optimal PSNR value in any of the experiments. According to STD, the proposed mSAR comes in the first position, followed by the GSA in the second position, while SCA and HHO come in the third position, the LFD is in the fourth position, and the original SAR comes in the fifth position. Finally, AOA and EO are ranked sixth.

Table 6 displays the mean and STD outcomes of SSIM for all test images, the optimal outcomes obtained are displayed in bold. It can be noticed that the mSAR displayed superiority in MTH after obtaining optimal SSIM values for 20 cases from 32 experiments. The original SAR comes in the second position with seven experiments, while SCA comes in the third position with five experiments, followed by HHO and EO in the fourth position with three experiments, and AOA in the fifth position with two experiments. Finally, without any experiments, GSA and LFD are in the last position. In terms of the value of STD, the original SAR is the best algorithm because its value is lower in the majority of experiments. The SCA is in the second position, followed by HHO and mSAR, while the AOA comes in the fourth position with the lower value of the STD. The LFD is in the fifth position, followed by the GSA. Finally, the EO comes in last with the least stable algorithm. Where *n* is the total number of pixels in image, μ is the mean of pixels, and $\sigma_{j}$ is the value of *j*th in the image.

In Table 7, the outcomes of the mean and STD values of FSIM obtained by the proposed mSAR and other competing algorithms are presented, and the optimal results are highlighted in bold. mSAR obtains the highest FSIM values and comes in the first position with 21 experiments, while SCA comes in second with six experiments, followed by the original SAR in the third position with five experiments. However, the HHO comes in the fourth position with four experiments, followed by the EO, which comes in the fifth position with only two experiments. Finally, LFD, GSA, and AOA cannot obtain the optimal FSIM value in any experiments. The mSAR algorithm is the best stable algorithm because its STD value is lower in most experiments, while the SAR comes in second, followed by the SCA algorithm. Then, GSA and HHO come in the fourth position, and LFD comes in the fifth position. Finally, the two most unstable algorithms are EO and AOA due to their high STD values in most experiments.

Finally, Table A2 in Appendix A illustrates the Wilcoxon rank-sum test for fitness using the fuzzy entropy method as an objective function. It is observed that there is a difference between the proposed mSAR algorithm and other competing algorithms during the execution of the Wilcoxon rank-sum test between mSAR and the following algorithms: LFD, HHO, SCA, EO, GSA, AOA, and SAR. This difference indicates that the proposed mSAR algorithm has undergone significant development. However, when compared to the EO, AOA, and SAR, it is clear that they have comparable behavior, as seen in the tables when (p>0.05) or NaN values occur.

### 5.5. Results Analysis of the Proposed Algorithm Based on the Otsu Method

**Figure 9:**
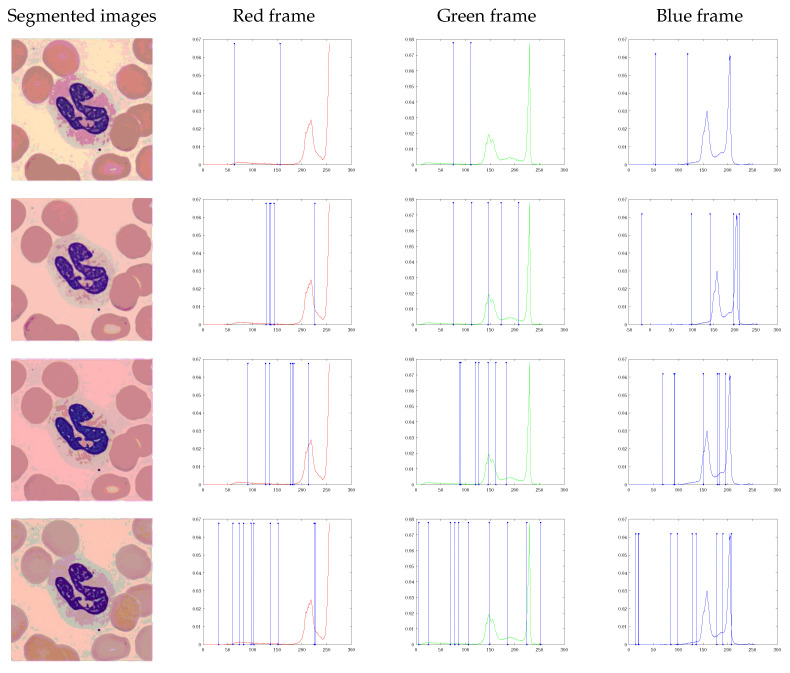
Threshold outcomes after executing mSAR on the Otsu method over the set of test images.

**Figure A2:**
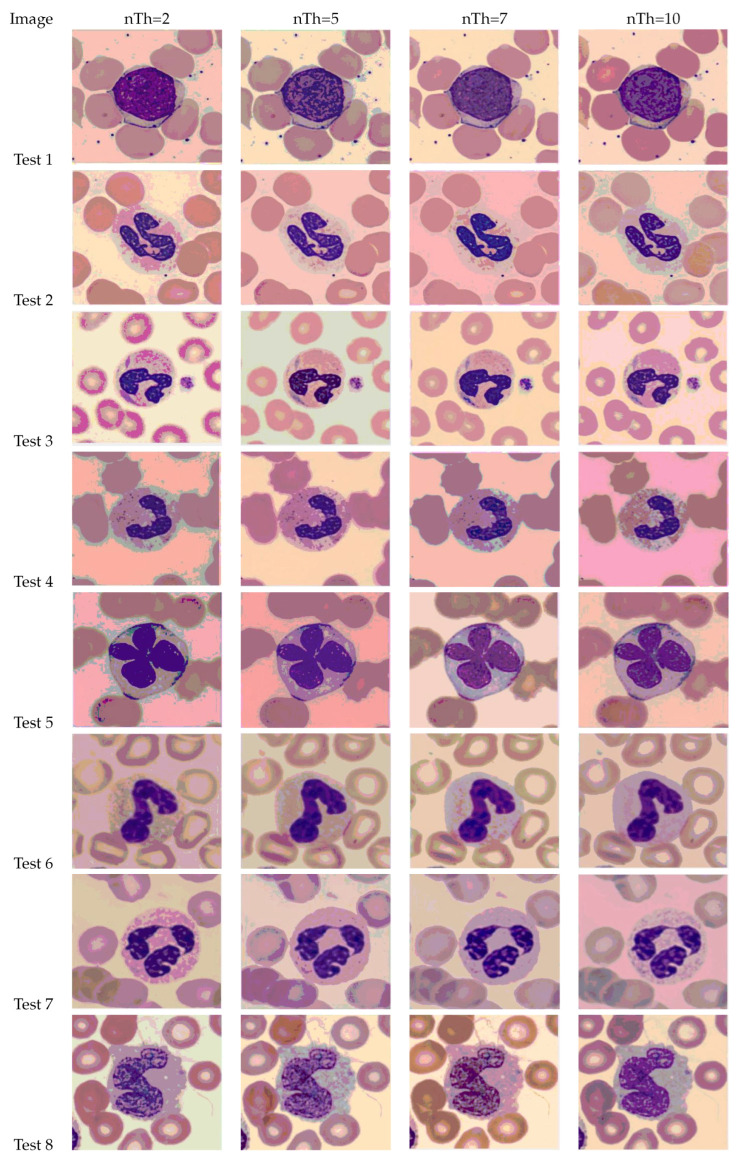
Outcomes after executing mSAR on the Otsu method over the set of test images.

This section discusses and analyzes the outcomes of the proposed mSAR algorithm using different numbers of thresholds [nT h = 2, 5, 7, 10]. The Otsu method is utilized in this subsection as an objective function for obtaining the best thresholds. The resulting images of the mSAR algorithm and other completed algorithms with various numbers of thresholds for all the test images used in the experiments are shown in Figure A2 in Appendix A. Figure 9 illustrates the resulting images of the mSAR with a different number of thresholds over test image 2, and shows a graphical analysis of the thresholds of the segmented images. Table A3 of Appendix A illustrates the optimal thresholding values obtained by the mSAR algorithm and the original SAR algorithm using the Otsu method. Table 8, Table 9, Table 10 and Table 11 illustrate the mean and STD outcomes of the fitness, PSNR, SSIM, and FSIM, respectively.

The mean and STD of fitness outcomes obtained by the mSAR algorithm and other completed algorithms for all test images are shown in Table 8. The proposed mSAR algorithm comes in the first position with 22 higher fitness values in 32 experiments, followed by the original SAR in the second position with seven experiments, and HHO in the third position with four experiments. However, GSA comes in at the fourth position with three experiments, and SCA obtains the fifth position with only two experiments. The remaining algorithms, LFD, EO, and AOA, finish last with no experiments. The values of STD computed for each of the benchmark images with a different number of thresholds are shown in Table 8, and the algorithm is less stable when the value of STD is higher. According to the values of the STD, HHO comes in the first position, while the mSAR and SAR come in the second position, followed by LFD. AOA comes in the fourth position, and the GSA takes the fifth position. The final algorithms, SCA and EO, come in the last position.

Table 9 illustrates the Mean and STD of the PSNR outcomes for all test images, the optimal outcomes obtained are highlighted in bold. It is noticed that the mSAR displayed superiority in MTH after obtaining optimal PSNR values for 17 cases from 32 experiments. The original SAR obtains the second position with nine experiments, while HHO comes in the third position with seven experiments, followed by SCA in the fourth position with six experiments. However, the AOA and EO rank fifth with only one experiment, while the GSA and LFD come in last with no experiments. According to the values of STD, LFD is in the first position, followed by GSA. However, the original SAR comes in third place, while mSAR comes in fourth position. AOA comes in the fifth position, while the SCA and HHO come in the sixth position. In the end, the EO comes in the last position with the highest value of STD and a less stable algorithm.

In Table 10, the outcomes of the mean and STD values of SSIM obtained by the proposed mSAR and other completed algorithms are presented, and the optimal results are noted in bold. The outcomes indicate that mSAR is in the first position for twenty-one cases from thirty-two experiments in terms of the SSIM. The HHO is in the second position with eleven experiments, while the original SAR comes in the third position with four experiments. However, the EO obtains the fourth position with three experiments, followed by the LFD in the fifth position with only two experiments. Finally, the remaining algorithms, SCA, GSA, and AOA, come in the last position, without any experiments. The original SAR and mSAR algorithms are the best stable algorithms because their STD values are lower in most experiments, while GSA comes in the second position followed by AOA. However, SCA and HHO obtain the fourth position, and LFD comes in the fifth position. Finally, due to its high STD values in the majority of experiments, EO is the most unstable algorithm.

The mean and STD of the FSIM outcomes for all test images are displayed in Table 11, and the optimal outcomes are highlighted in bold. According to the outcomes of the FSIM, the EO comes in the first position with fifteen cases from thirty-two experiments, followed by mSAR in the second position with thirteen higher cases. HHO comes in the third position with six experiments, while the LFD is in the fourth position with five experiments. However, the original SAR obtained the fifth position with four experiments, and the SCA came in the sixth position with only three experiments. Finally, GSA and AOA come in last, without any experiments. According to the STD values, GSA comes in the first position, followed by the original SAR and mSAR in the second position. However, LFD obtains the third position, and AOA comes in the fourth position. EO comes in the fifth position, while the remaining algorithms, SCA and HHO, come in last, with less stable algorithms.

The outcomes of the Wilcoxon rank-sum test for fitness using the Otsu method as an objective function are shown in Table A4 of Appendix A. During execution of the Wilcoxon rank-sum test between mSAR and the following algorithms (LFD, HHO, SCA, EO, GSA, AOA, and SAR), it was noticed that there was a difference between the proposed mSAR algorithm and the other competing algorithms. This difference indicates that the proposed mSAR algorithm has undergone significant development. However, when compared to the EO and SAR, it is clear that they have comparable behavior, as noticed in tables with (p>0.05) or NaN values. According to previous results, the results of the mSAR algorithm in the Fuzzy Entropy method are better than the results of the Otsu method, especially in nTh=7. Additionally, the quality of segmented color images using the fuzzy entropy method is high, as shown in Figure 8.

### 5.6. The Pros and Cons of the mSAR Algorithm

This subsection discusses the benefits and downsides of the proposed mSAR algorithm. The main benefits of the proposed mSAR can be summarized as its ease of implementation and understanding. The proposed mSAR algorithm has proven superior in solving color image-segmentation problems when compared to competing algorithms and gives good outcomes in terms of the quality of the segmented images.Furthermore, the mSAR produces a high convergence speed, indicating that the mSAR avoids entrapping in local optima and successfully balances the interchange between exploration and exploitation phases, owing to the fast finding of the threshold values and the high accuracy of the outcomes. The main defect of the mSAR is that the proposed algorithm is simple but computationally expensive. Additionally, the outcomes of STD values are not good enough to compete with the other algorithms.

## 6. Conclusions and Future Work

This paper presents an improved version of the SAR algorithm utilizing the concept of the OBL technique called mSAR. OBL improves the searchability of the original SAR, enables it to avoid entrapping in local optima, and successfully balances between the exploration and exploitation phases. mSAR is utilized to segment blood cell images and solve the problems of multi-level thresholding for image segmentation. IEEE CEC’2020 benchmark functions are utilized to prove the sturdiness of mSAR, and the proposed mSAR is implemented on a real application and executed as a multi-level image segmentation tool for estimating its performance on a set of color blood images. The proposed algorithm utilized fuzzy entropy and Otsu methods as objective functions to determine the optimal thresholds during the segmentation processes. The outcomes of the experiment proved the superiority of the proposed mSAR algorithm in producing good segmentation performance to segment the blood images and solve the CEC’2020 benchmark functions compared with other optimization algorithms. In future work, the proposed mSAR can be utilized to track the size of blood cells and tackle different problems such as feature selection, parameter identification, and task scheduling.

## Figures and Tables

**Table 1:** CEC’2020 benchmark functions.

No.	Function Description	Fi*
**Unimodal function**		
F1	Shifted and Rotated Bent Cigar Function	100
**Multimodal shifted and rotated functions**		
F2	Shifted and Rotated Schwefel’s Function	1100
F3	Shifted and Rotated Lunacek bi-Rastrigin Function	700
F4	Expanded Rosenbrock’s plus Griewangk’s Function	1900
**Hybrid functions**		
F5	Hybrid Function 1 (N = 3)	1700
F6	Hybrid Function 2 (N = 4)	1600
F7	Hybrid Function 3 (N = 5)	2100
**Composition functions**		
F8	Composition Function 1 (N = 3)	2200
F9	Composition Function 2 (N = 4)	2400
F10	Composition Function 3 (N = 5)	2500

**Table 2:** STD and mean of the optimum value obtained from the proposed mSAR algorithm against other competing algorithms on the CEC’2020 benchmark functions with D=20.

Functions	LFD		HHO		SCA		EO		GSA		AOA		SAR		mSAR	
	Mean	Std	Mean	Std	Mean	Std	Mean	Std	Mean	Std	Mean	Std	Mean	Std	Mean	Std
F1	2.583E+10	3.262E+06	4.733E+06	1.350E+05	8.256E+09	3.102E+04	**2.307E+03**	1.304E+09	4.593E+09	4.652E+03	3.398E+09	3.130E+03	1.427E+06	3.560E+01	3.942E+08	1.418E+07
F2	4.647E+03	3.201E+02	3.032E+03	2.970E+04	4.892E+03	4.028E+03	4.220E+03	6.356E+03	4.685E+04	6.301E+01	4.084E+03	4.264E+02	3.236E+03	2.003E+02	**2.496E+03**	2.537E+03
F3	1.056E+03	2.040E+01	9.340E+02	6.623E+01	9.376E+02	3.601E+01	7.478E+02	2.630E+01	8.686E+02	2.655E+03	8.779E+02	6.120E+03	7.721E+02	3.784E+05	**1.077E+02**	6.835E+02
F4	1.636E+05	8.611E+00	1.919E+03	1.301E+03	3.277E+03	5.103E+00	**1.903E+03**	9.319E+03	6.343E+04	2.368E+06	2.472E+03	1.442E+00	1.906E+03	2.230E+01	6.471E+03	5.651E+05
F5	4.908E+06	3.120E+04	6.073E+04	6.310E+04	1.152E+06	7.214E+04	1.454E+05	6.531E+01	8.328E+07	3.791E+02	1.525E+06	1.681E+02	5.776E+05	6.552E+03	**1.447E+04**	7.658E+03
F6	1.667E+03	2.119E+05	1.857E+03	2.014E+02	2.049E+03	1.620E+01	1.624E+03	9.300E+01	1.624E+03	6.240E+02	2.371E+03	0.000E+00	2.049E+03	3.567E+03	**1.883E+02**	4.681E+01
F7	1.891E+06	9.101E+07	3.251E+05	7.302E+01	2.718E+05	2.531E+05	9.194E+04	3.613E+04	2.144E+06	0.000E+00	1.745E+05	6.067E+03	3.955E+03	0.000E+00	**1.056E+03**	3.597E+02
F8	5.111E+03	4.715E+03	**2.305E+03**	5.200E+00	3.165E+03	1.435E+03	2.597E+03	7.020E+03	4.968E+04	6.234E+01	5.021E+03	2.641E+05	2.311E+03	6.302E+06	6.491E+03	0.000E+00
F9	3.022E+03	2.605E+06	3.258E+03	4.106E+04	2.975E+03	6.165E+02	2.827E+03	5.460E+02	3.533E+03	1.563E+06	2.985E+03	6.340E+04	2.910E+03	3.409E+02	**1.004E+03**	3.564E+03
F10	4.505E+03	3.106E+03	3.005E+03	5.780E+03	3.220E+03	3.031E+03	2.931E+04	3.631E+00	3.310E+05	2.061E+02	3.162E+03	5.987E+02	2.913E+03	1.613E+05	**2.139E+03**	5.651E+04
Friedman mean rank	6.60		3.80		5.55		2.95		6.75		5.00		2.95		2.40	
Rank	7		4		6		2		8		5		3		1	

**Table 3:** The parameter settings of the mSAR algorithm and its value.

Parameter	Value
Maximum iterations $\left(T_{\max}\right)$	350
Number of independent runs (t)	30
Population size (N)	50
Dimension of problem (Dim)	20
Mutation ratio (P)	0.5
Social effect parameter (SE)	0.05

**Table 4:** mSAR fuzzy entropy in terms of fitness values over all competed algorithms.

		LFD		HHO		SCA		EO		GSA		AOA		SAR		mSAR	
Test Image	nTh	Mean	Std	Mean	Std	Mean	Std	Mean	Std	Mean	Std	Mean	Std	Mean	Std	Mean	Std
Test 1	2	18.1046	2.8080E-16	18.0759	2.8440E-16	18.1062	2.7720E-16	18.0876	2.9991E-04	18.0261	2.7360E-16	18.0082	1.9760E-02	**18.2110**	2.5560E-16	18.1255	2.4120E-16
	5	22.6431	2.8080E-16	22.6264	2.8440E-16	22.6153	3.9963E-03	22.5722	2.1917E-03	22.5199	1.3756E-03	22.6169	2.7360E-16	**22.7893**	2.5560E-16	22.6808	2.4120E-16
	7	27.3154	1.9500E-04	27.3259	8.5320E-16	27.3560	1.1935E-03	27.0981	4.7140E-03	27.1008	1.1400E-04	27.3199	1.7480E-04	27.3184	7.6680E-16	**27.3903**	7.2360E-16
	10	31.3638	1.9500E-04	31.4052	1.5642E-03	31.4301	2.1637E-03	31.0209	7.9976E-03	31.1137	5.0236E-03	31.3801	3.5796E-03	31.4205	2.5560E-16	**31.5391**	2.4120E-16
Test 2	2	18.3414	2.8080E-16	18.3139	2.8440E-16	18.3425	2.7720E-16	18.3344	1.3073E-04	18.2793	2.6752E-15	18.3072	2.7360E-16	18.3491	2.5560E-16	**18.3964**	2.4120E-16
	5	22.9591	2.7066E-15	**23.7630**	9.4010E-03	22.8547	9.2400E-03	**23.7630**	2.8684E-03	22.8249	6.3916E-03	22.7600	7.3188E-03	23.1217	2.5560E-16	23.0566	2.4120E-16
	7	27.3599	1.2246E-05	27.3536	1.2087E-05	27.3652	8.5470E-03	27.4435	5.2061E-03	27.1778	4.6436E-05	27.1115	2.3940E-02	27.4506	1.0224E-15	**27.4735**	9.6480E-16
	10	31.3065	4.0560E-04	31.3372	8.6900E-04	31.3677	8.1620E-04	**31.4664**	1.0151E-02	31.1128	1.0412E-03	31.1385	2.5916E-02	31.3560	1.2780E-15	**31.4664**	1.2060E-15
Test 3	2	18.1695	2.8080E-16	18.1460	2.8440E-16	18.1701	2.7720E-16	18.1662	6.4135E-05	18.1142	8.1320E-15	18.1351	2.7360E-16	18.1700	2.5560E-16	**18.2409**	2.4120E-16
	5	22.7428	3.3306E-03	22.7042	1.8170E-03	22.8149	1.0318E-05	22.6968	4.4602E-04	22.6584	3.1616E-03	22.6843	1.3680E-15	22.7794	2.9465E-03	**22.8716**	2.7805E-03
	7	27.2930	2.8860E-04	27.1417	1.5642E-02	27.3592	3.6190E-04	26.9526	7.0979E-03	26.9972	1.4668E-02	26.9902	1.1172E-02	27.3330	5.1191E-16	**27.4420**	4.8307E-16
	10	31.4630	8.2680E-04	31.4970	1.5326E-02	31.5696	7.0070E-04	30.8451	1.3996E-02	31.1545	2.0900E-02	31.1630	2.5536E-02	31.5604	7.6680E-16	**31.6866**	7.2360E-16
Test 4	2	18.4294	1.1232E-15	18.4057	1.1376E-15	**18.4300**	2.4178E-07	18.4208	1.9994E-04	18.3649	1.0944E-15	18.3944	1.0944E-15	18.4295	1.0224E-15	18.4020	1.0656E-15
	5	23.2453	2.6208E-06	**23.2518**	8.5320E-16	23.2511	9.2400E-05	23.1999	9.3818E-04	23.1441	8.2080E-16	23.2088	8.2080E-16	**23.2518**	7.6680E-16	23.2427	7.9920E-16
	7	27.5700	2.7300E-05	27.5607	3.8789E-05	**27.5948**	3.3572E-05	27.4411	2.6223E-03	27.4436	1.1400E-04	27.5443	1.4136E-05	27.5907	2.5560E-16	27.5619	2.6640E-16
	10	31.6329	2.9718E-03	31.6810	4.8980E-03	**31.7427**	1.1319E-03	**31.7427**	5.2907E-03	31.4583	6.2548E-03	31.5898	5.7608E-03	31.7314	2.5560E-16	31.0452	2.6640E-16
Test 5	2	18.1587	2.8080E-16	18.1330	2.8440E-16	18.1594	2.7720E-16	18.1546	6.8133E-05	18.0990	2.1584E-15	18.1242	2.7360E-16	18.1766	2.5560E-16	**18.3358**	2.6640E-16
	5	23.0376	5.7408E-15	23.0165	8.5320E-16	23.0426	2.1637E-03	22.9820	1.2919E-03	22.9274	9.8800E-15	23.0065	8.2080E-16	23.0698	7.6680E-16	**23.2735**	7.9920E-16
	7	27.6085	1.6380E-04	27.4755	2.1251E-02	27.6783	1.2782E-02	27.0664	1.2996E-02	27.3479	1.5580E-02	27.3909	2.0520E-02	27.7290	1.2780E-15	**27.9806**	1.3140E-15
	10	31.6238	2.2074E-03	31.7292	2.0935E-02	31.8116	1.0626E-02	30.7854	1.7918E-02	31.3514	2.1660E-02	31.4811	2.3712E-02	**31.8731**	7.6680E-06	31.8507	2.6280E-16
Test 6	2	18.7103	5.6238E-16	18.6849	5.6959E-16	18.7108	5.5517E-16	18.7053	1.1535E-04	18.6505	5.4796E-16	18.6745	5.4796E-16	18.7088	5.1191E-16	**18.7111**	5.2633E-16
	5	23.2793	1.3260E-04	23.2549	1.0428E-05	23.2864	2.2330E-04	23.2476	9.2280E-04	23.1915	1.9760E-04	23.2411	3.6176E-06	23.2909	2.3430E-04	**23.2886**	2.6280E-04
	7	27.7771	4.9218E-05	27.7951	5.6485E-03	**27.8501**	6.8838E-05	27.5574	5.4215E-03	27.6000	5.4872E-03	27.6920	9.5760E-03	27.8342	1.2780E-15	**27.8501**	1.3140E-15
	10	31.8757	4.6020E-04	31.9350	4.1870E-04	31.9670	1.1858E-03	**31.9953**	8.4590E-03	31.6525	1.1096E-03	31.8810	2.4472E-03	31.9853	0.0000E+00	31.9701	0.0000E+00
Test 7	2	18.4319	2.8080E-16	18.4087	1.8802E-05	18.4334	1.1242E-05	18.4237	2.6915E-04	18.3675	1.6188E-05	18.3981	8.5880E-06	18.4509	2.5560E-16	**18.5410**	2.7360E-16
	5	23.0903	8.4240E-16	23.0759	6.1462E-05	23.1040	1.6940E-04	23.0421	1.1381E-03	22.9856	1.2160E-04	23.0633	8.2080E-16	23.1266	7.6680E-16	**23.2402**	8.2080E-16
	7	27.3927	7.8000E-05	27.3945	6.0040E-04	27.4277	6.0830E-04	27.2754	3.2375E-03	27.2679	4.8640E-04	27.3820	3.9520E-04	27.4576	1.7892E-15	**27.5938**	1.9152E-15
	10	31.3324	4.3680E-04	31.3642	5.6880E-04	31.3989	1.2551E-03	31.0497	6.7903E-03	31.1869	9.1960E-04	31.3404	1.1020E-03	31.4269	2.0448E-15	**31.5843**	1.9872E-15
Test 8	2	18.3020	1.1232E-15	18.2744	1.1376E-15	18.3069	7.9310E-06	18.1867	9.9970E-04	18.1901	1.0944E-15	18.0382	3.2604E-02	**18.3211**	1.0224E-15	**18.3211**	9.9360E-16
	5	23.0376	5.7408E-05	23.0578	8.5320E-16	22.9703	1.3552E-02	22.7504	5.9444E-03	**23.1041**	2.3560E-04	23.0521	8.2080E-16	**23.1041**	7.6680E-16	23.0448	7.4520E-16
	7	27.1252	1.4820E-04	27.1816	2.6781E-05	27.1951	3.8808E-03	26.7231	9.2280E-03	26.8257	2.2040E-04	27.1838	1.3680E-04	27.2266	7.6680E-16	**27.3973**	7.4520E-16
	10	30.8359	2.0436E-03	30.9707	1.5484E-03	31.0086	2.7258E-03	29.9156	5.1062E-02	30.5448	1.8316E-03	30.8748	1.5732E-02	31.0187	5.1191E-16	**31.2114**	4.9749E-16

**Table 5:** mSAR fuzzy entropy in terms of PSNR values over all competed algorithms.

		LFD		HHO		SCA		EO		GSA		AOA		SAR		mSAR	
Test Image	nTh	Mean	Std	Mean	Std	Mean	Std	Mean	Std	Mean	Std	Mean	Std	Mean	Std	Mean	Std
Test 1	2	14.0348	5.5380E-14	14.0484	4.6150E-14	**14.1710**	6.4610E-13	14.02757	5.3520E-02	13.9516	4.6150E-13	14.0100	5.5380E-13	14.0820	9.7308E-12	**14.1710**	4.4304E-13
	5	14.8938	2.2700E-04	14.9083	8.3000E-04	**16.7024**	3.9900E-03	15.04624	3.3600E-01	12.4478	2.3050E-13	14.8360	0.0000E+00	14.9227	5.8428E-12	15.0384	2.2128E-13
	7	20.7545	1.6860E-03	20.7777	2.0200E-03	**20.9695**	2.4430E-02	20.22302	1.1340E+01	12.9449	1.1550E-12	20.6739	5.5800E-03	20.7979	0.0000E+00	20.9591	1.1088E-12
	10	21.2726	4.2840E-03	21.3499	2.3050E-03	21.6258	6.8600E-03	20.89767	8.6400E+00	13.7842	6.7500E-01	21.2618	7.3800E-03	**21.6922**	1.5552E-11	21.4874	1.1088E-12
Test 2	2	15.0771	4.1520E-14	15.0918	3.4600E-14	15.0235	4.8440E-13	15.07096	4.9500E-02	14.0941	3.4600E-13	15.0186	4.1520E-13	15.1064	5.8428E-12	**15.2771**	3.3216E-13
	5	16.7056	5.5380E-14	17.2239	1.2500E-03	17.5063	6.4610E-13	16.70248	4.9200E-01	12.1618	4.6150E-13	**17.5733**	2.7660E-13	16.7380	7.7868E-12	16.7056	4.4304E-13
	7	19.8656	1.1940E-03	19.8849	2.0700E-03	20.0460	1.0500E+01	19.42992	1.7580E+01	15.7123	2.3050E-13	19.7146	8.2800E-03	19.9042	3.8880E-12	**20.0656**	2.2128E-13
	10	21.5301	3.6360E-01	21.5098	3.4100E-02	21.6965	5.1450E+00	20.87707	1.2300E+01	17.5084	2.8900E-01	21.1859	4.6140E-01	21.5595	1.9440E-11	**21.7431**	0.0000E+00
Test 3	2	16.4975	0.0000E+00	16.5135	0.0000E+00	16.6577	0.0000E+00	**16.7577**	7.2000E-02	14.1762	0.0000E+00	16.4334	0.0000E+00	16.5295	3.8880E-12	**16.7577**	0.0000E+00
	5	18.1259	2.4000E-04	19.1838	2.6550E-03	16.6577	4.8440E-13	19.1889	1.7340E+01	15.9442	3.4600E-13	**19.2519**	1.7400E-03	17.1444	1.2639E+03	17.2442	3.3216E-13
	7	19.1467	9.6000E-04	20.1591	3.1100E-03	19.4750	1.2040E-02	18.85518	1.4340E+01	15.3015	1.4450E-04	**20.6370**	5.4600E-03	19.3269	0.0000E+00	19.4395	0.0000E+00
	10	21.0007	3.2400E-03	21.2561	2.6650E-02	21.2930	3.0310E+00	19.6112	1.2120E+01	15.4606	1.1500E-03	21.8600	3.6000E-02	21.0666	7.7868E-12	**21.8624**	3.3216E-13
Test 4	2	15.6581	8.2800E-14	15.6733	6.9000E-14	15.8101	9.6600E-13	15.63334	2.5200E-02	15.6291	6.9000E-13	15.5973	5.5380E-13	15.6581	9.7308E-12	**15.8493**	6.6240E-13
	5	19.0529	7.8000E-05	19.0714	1.1850E-03	19.2348	1.6170E-12	18.96642	3.4560E-01	15.8850	1.1550E-12	18.9789	8.2800E-13	19.0529	3.8880E-12	**19.3348**	1.1088E-12
	7	21.6650	2.5140E-03	21.6840	3.4150E-03	21.8681	1.0570E-02	21.38692	9.4200E-01	17.4491	4.5500E-03	21.5768	4.4520E-02	**21.8788**	7.7868E-12	21.7850	2.2128E-13
	10	21.6908	5.0340E-03	22.0469	5.8000E-03	21.8826	9.3100E-03	21.99977	1.1460E+01	18.6670	3.0450E-04	**22.7300**	2.1060E-02	21.7206	0.0000E+00	21.8472	2.2128E-13
Test 5	2	16.2307	5.5380E-14	**16.4465**	4.0000E-04	16.3883	6.4610E-13	16.27194	2.2140E-02	12.6162	4.6150E-13	16.1677	2.7660E-13	16.0732	9.7308E-12	**16.4465**	4.4304E-13
	5	19.3743	4.8000E-05	19.3931	2.1000E-03	**19.4740**	0.0000E+00	19.18993	3.3840E-01	13.1726	0.0000E+00	19.2991	5.0940E-04	19.1862	3.8880E-12	19.3179	0.0000E+00
	7	19.3671	7.5000E-04	**20.0715**	3.7300E-03	19.5853	3.5000E-03	19.19302	1.1040E+01	12.8097	2.3050E-13	20.0101	4.4400E-03	19.1862	3.8880E-12	19.3179	2.2128E-13
	10	20.2951	2.6400E-01	21.0314	4.0000E-03	20.8458	4.2910E+00	19.53292	8.8200E+00	12.6589	1.8500E-03	20.8432	1.0140E-02	20.7274	1.3364E-01	**21.0707**	2.2128E-13
Test 6	2	16.7530	0.0000E+00	16.7692	0.0000E+00	16.9156	2.5480E-02	16.75604	5.6400E-02	14.5870	0.0000E+00	16.6879	0.0000E+00	16.5903	3.8880E-12	**16.9642**	0.0000E+00
	5	18.6090	0.0000E+00	18.9302	2.0150E-05	18.4766	1.1900E-03	18.68523	1.4640E+01	15.1882	0.0000E+00	18.8435	0.0000E+00	17.0870	9.6324E+02	**19.0061**	0.0000E+00
	7	18.9087	6.0000E-04	19.0137	2.3000E-04	19.1100	3.1920E-02	18.64094	1.7400E+01	13.7124	4.9900E-04	19.0518	8.2800E-13	**19.8905**	0.0000E+00	**19.8905**	0.0000E+00
	10	21.1511	4.6560E-03	21.2664	8.7500E-03	20.9799	3.1220E-02	20.1056	1.4520E+01	15.7414	4.1500E-03	20.8606	1.9740E-02	21.4048	7.7868E-12	**21.3012**	4.4304E-13
Test 7	2	15.0339	9.6600E-14	15.0629	8.0500E-14	**15.1746**	1.1270E-12	14.97929	5.3460E-02	14.2698	8.0500E-13	14.9775	9.6600E-13	15.0777	5.8428E-12	15.0047	7.7280E-13
	5	17.6604	5.5380E-14	17.6868	1.8950E-05	**17.8859**	1.3300E-03	17.79222	1.2960E+01	14.5278	4.6150E-13	17.5918	8.2800E-13	**19.8905**	3.8880E-12	17.6261	4.4304E-13
	7	19.6988	9.4800E-04	19.7725	2.6650E-03	19.7153	3.9620E-02	19.91402	1.6560E+01	14.6193	5.7500E-13	19.6417	5.5380E-13	19.6992	3.8880E-12	**19.9289**	5.5200E-13
	10	21.7371	2.8980E-03	21.7809	2.1150E-02	21.8202	3.7380E-02	21.10676	1.4220E+01	15.9411	2.2500E-03	21.4732	1.7220E-02	21.8230	1.1664E-11	**22.8775**	4.4304E-13
Test 8	2	12.6124	4.1520E-14	12.6246	3.4600E-14	12.7046	4.8440E-13	12.51038	3.6600E-02	12.6225	3.4600E-13	13.3277	4.1520E-13	12.6001	1.9440E-12	**13.7470**	3.3216E-13
	5	17.3823	8.2800E-14	17.4012	6.9000E-14	17.1725	9.8700E-02	16.98058	5.7600E+00	14.9760	6.9000E-13	17.3168	4.1520E-13	17.3675	0.0000E+00	**17.5700**	6.6240E-13
	7	20.6803	2.6040E-01	**20.9314**	1.0100E-03	20.7875	4.4240E-02	19.79248	9.9600E+00	15.8194	6.3500E-03	20.6093	6.9000E-13	20.6901	7.7868E-12	**20.9314**	4.4304E-13
	10	21.4920	2.2620E-01	21.8764	9.8000E-03	21.6746	2.6460E+00	21.15723	6.8400E+00	16.0368	1.2500E-03	21.7091	5.6400E-03	20.6901	7.7868E-12	**21.9314**	3.3216E-13

**Table 6:** mSAR fuzzy entropy in terms of SSIM values over all competed algorithms.

		LFD		HHO		SCA		EO		GSA		AOA		SAR		mSAR	
Test Image	nTh	Mean	Std	Mean	Std	Mean	Std	Mean	Std	Mean	Std	Mean	Std	Mean	Std	Mean	Std
Test 1	2	0.6904	3.4727E-15	0.6911	3.1570E-15	0.6751	3.4276E-15	0.6973	1.1529E-02	0.6921	3.2923E-14	**0.6991**	3.1119E-14	0.6990	3.1254E-15	**0.6991**	4.5100E-15
	5	0.7255	4.4044E-04	0.7262	5.8240E-03	0.7094	6.5512E-04	0.7318	5.9749E-02	0.7273	5.7597E-14	0.7345	5.4441E-14	0.7345	5.4678E-15	**0.7355**	5.6019E-15
	7	0.7471	3.5343E-03	0.7478	3.8570E-03	0.7304	2.9792E-03	0.7509	4.5494E-02	0.7489	3.2923E-14	0.7563	1.5387E-02	**0.7564**	3.1254E-15	**0.7564**	3.2021E-15
	10	0.7713	7.2534E-03	0.7721	6.0900E-03	0.7542	4.0280E-03	0.7682	6.9565E-02	0.7732	1.4892E-01	0.7809	2.4909E-02	0.7710	2.3423E-15	**0.7823**	2.3998E-15
Test 2	2	0.5808	8.7010E-16	0.5813	7.9100E-16	0.5678	8.5880E-16	0.5875	1.6281E-02	0.5822	8.2490E-15	0.5879	7.7970E-15	**0.5880**	7.8309E-16	0.5815	8.0230E-16
	5	0.6491	3.4727E-15	0.6497	8.5400E-03	**0.6572**	3.4276E-15	0.6547	5.1959E-02	0.6507	3.2923E-14	0.6571	3.1119E-14	**0.6572**	3.1254E-15	0.6499	3.2021E-15
	7	0.6877	1.0934E-03	0.6884	1.2250E-03	0.6707	4.8260E-02	0.6828	1.6515E-01	0.6894	3.2923E-14	**0.6968**	7.0380E-03	0.6963	3.1254E-15	**0.6968**	3.2021E-14
	10	0.7093	7.2534E-02	**0.7272**	7.6300E-02	0.6918	6.9768E-02	0.7001	1.5113E-01	0.7119	6.4751E-01	0.7136	8.4180E-01	0.7154	1.5593E-15	**0.7272**	1.5975E-15
Test 3	2	0.7093	8.7010E-16	0.7100	7.9100E-16	0.6935	8.5880E-16	0.7164	1.8462E-02	0.7110	8.2490E-15	0.7181	7.7970E-15	**0.7182**	7.8309E-16	0.7181	8.0230E-16
	5	0.7677	3.2340E-04	0.7694	1.0990E-02	0.7507	5.9964E-15	0.7700	1.6904E-01	0.7696	5.7597E-14	0.7772	2.0769E-03	0.7773	5.4678E-15	**0.7778**	5.6019E-14
	7	0.8217	6.2986E-03	0.8216	8.0500E-03	**0.8425**	7.1136E-03	0.8118	2.0799E-01	0.8219	2.4017E-02	0.8309	5.0508E-02	0.8301	2.3423E-15	0.8300	2.3998E-15
	10	0.8397	9.1630E-03	0.8414	1.7710E-02	**0.8501**	2.6448E-02	0.8317	1.9241E-01	0.8408	4.9932E-03	0.8500	1.0902E-01	0.8492	3.9016E-15	**0.8501**	3.9973E-15
Test 4	2	0.6814	1.7325E-15	0.6821	1.5750E-15	0.6663	1.7100E-15	0.6883	6.8630E-03	0.6831	1.6425E-14	0.6899	1.5525E-14	0.6899	1.5593E-15	**0.6909**	1.5975E-15
	5	0.7615	2.9876E-03	0.7622	7.4200E-03	0.7445	4.2788E-15	0.7691	2.2981E-02	0.7633	4.1099E-14	0.7709	3.8847E-14	0.7709	3.9016E-15	**0.7809**	3.9973E-15
	7	0.7983	1.4784E-02	0.8000	2.2540E-02	0.7788	5.0008E-03	0.8090	5.6010E-02	0.7985	2.7083E-02	0.8091	1.9389E-01	0.8064	2.3423E-15	**0.8363**	2.3998E-15
	10	0.8289	2.9722E-03	**0.8498**	2.9610E-03	0.8104	1.7404E-03	0.8217	1.2542E-01	0.8309	4.9129E-03	0.8391	8.9700E-03	0.8392	3.9016E-15	**0.8498**	3.9973E-16
Test 5	2	0.7543	1.7325E-15	0.7550	2.9540E-03	0.7375	1.7100E-15	0.7627	1.2230E-02	0.7561	1.6425E-14	0.7636	1.5525E-14	**0.7637**	1.5593E-14	0.7636	1.5975E-16
	5	0.8001	1.2782E-02	0.8027	2.5970E-02	0.7814	1.7100E-15	**0.8272**	9.1922E-02	0.8012	1.6425E-14	0.8000	1.2144E-01	0.8092	1.5593E-14	0.8001	1.5975E-16
	7	0.8190	6.4141E-03	0.8198	8.7500E-03	0.7999	4.1420E-03	**0.8436**	9.3480E-02	0.8201	3.2923E-14	0.8282	1.9389E-02	0.8283	3.1254E-14	0.8282	3.2021E-16
	10	0.8487	3.9193E-02	0.8504	5.0120E-03	**0.8698**	6.1484E-02	0.8372	1.2075E-01	0.8525	3.5916E-02	0.8610	3.8226E-02	0.8611	7.8309E-15	0.8610	8.0230E-17
Test 6	2	0.6823	2.6026E-15	0.6830	2.3660E-15	0.6672	2.1356E-03	0.6892	1.0828E-02	0.6840	2.4674E-14	0.6908	2.3322E-14	**0.6909**	2.3423E-14	0.6831	2.3998E-16
	5	0.7183	0.0000E+00	0.7190	1.7920E-04	0.7023	4.3700E-03	0.7246	5.5075E-02	0.7201	0.0000E+00	0.7272	0.0000E+00	0.7273	0.0000E+00	**0.7372**	0.0000E+00
	7	0.7740	9.7020E-04	0.7748	5.1170E-04	0.7568	3.0476E-03	0.7609	2.1968E-01	0.7759	1.3213E-03	0.7836	0.0000E+00	0.7837	0.0000E+00	**0.7936**	0.0000E+00
	10	0.7947	2.9953E-03	**0.8055**	3.8360E-03	0.7770	1.2008E-03	0.7845	1.4879E-01	0.7967	6.1539E-03	0.8045	1.6008E-02	0.8046	3.9016E-15	**0.8055**	3.9973E-15
Test 7	2	0.6572	0.0000E+00	0.6578	0.0000E+00	0.6425	0.0000E+00	**0.6657**	9.1922E-03	0.6588	0.0000E+00	0.6653	0.0000E+00	0.6654	0.0000E+00	0.6653	0.0000E+00
	5	0.7264	1.7325E-15	0.7271	1.3860E-03	0.7094	3.2528E-03	0.7291	1.0283E-01	0.7282	1.6425E-14	0.7354	1.5525E-14	0.7355	1.5593E-15	**0.7384**	1.5975E-15
	7	0.7965	7.1841E-03	0.7973	6.6010E-03	0.7788	1.2160E-02	0.7863	2.5473E-01	0.7985	4.1099E-14	0.8063	3.8847E-14	0.8064	3.9016E-15	0.8063	3.9973E-16
	10	0.8280	1.1165E-02	0.8270	1.0360E-02	**0.8378**	1.1096E-02	0.8154	1.8151E-01	0.8273	4.0223E-03	0.8355	6.6378E-02	0.8356	0.0000E+00	**0.8378**	0.0000E+00
Test 8	2	0.6302	3.4727E-15	0.6308	3.1570E-15	0.6162	3.4276E-15	0.6356	9.5038E-03	0.6317	3.2923E-14	0.6380	3.1119E-14	0.6381	3.1254E-15	**0.6388**	3.2021E-16
	5	0.7938	1.7325E-15	0.7946	1.5750E-15	0.7762	2.1052E-02	0.7891	3.7003E-01	0.7958	1.6425E-14	0.8036	1.5525E-14	0.8037	1.5593E-15	**0.8055**	1.5975E-15
	7	0.8253	1.6401E-01	0.8297	4.8440E-03	0.8104	1.5352E-02	0.7654	5.7101E-01	0.8318	3.1025E-02	0.8400	7.7970E-15	**0.8401**	7.8309E-16	0.8400	8.0230E-16
	10	0.8451	2.0251E-02	0.8477	2.6320E-03	0.8263	3.6404E-02	0.8290	3.1238E-01	0.8489	1.7885E-02	0.8573	2.6565E-02	0.8574	3.9016E-15	**0.8878**	3.9973E-15

**Table 7:** mSAR fuzzy entropy in terms of FSIM values over all competed algorithms.

		LFD		HHO		SCA		EO		GSA		AOA		SAR		mSAR	
Test Image	nTh	Mean	Std	Mean	Std	Mean	Std	Mean	Std	Mean	Std	Mean	Std	Mean	Std	Mean	Std
Test 1	2	0.7866	2.6702E-15	0.7889	2.6702E-15	0.7928	2.6364E-15	0.7834	1.5300E-02	0.7875	2.5688E-15	0.7326	2.1970E-14	**0.7989**	2.7040E-15	0.7889	2.6702E-16
	5	0.8310	1.2482E-04	0.8335	2.3321E-03	0.8376	1.8564E-04	0.8275	3.3240E-02	0.8320	1.7100E-15	0.7740	1.4625E-14	0.8335	1.8000E-15	**0.8441**	1.7775E-16
	7	0.8636	7.1600E-03	0.8667	5.2029E-03	0.8711	5.9670E-03	0.8544	9.7016E-02	0.8656	4.2788E-15	0.8052	2.1065E-02	**0.8782**	4.5040E-15	0.8672	4.4477E-16
	10	0.9040	4.4249E-03	0.9068	2.3487E-03	0.9114	3.6386E-03	0.8767	1.3644E-01	0.9048	1.9029E-02	0.8420	1.8084E-02	0.9182	6.3120E-15	**0.9218**	6.2331E-15
Test 2	2	0.7124	2.6702E-15	0.7145	2.6702E-15	0.7180	2.6364E-15	0.7110	1.1594E-02	0.7132	2.5688E-15	0.6635	2.1970E-14	0.7236	2.7040E-15	**0.7263**	2.6702E-15
	5	0.7687	1.7775E-15	0.7712	6.0727E-03	0.7748	1.7550E-15	0.7660	4.0945E-02	0.7696	1.7100E-15	0.7160	1.4625E-14	0.7808	1.8000E-15	**0.7838**	1.7775E-15
	7	0.8196	2.3324E-03	0.8218	3.6293E-03	0.8232	8.5020E-02	0.7960	1.8010E-01	0.8207	2.5688E-15	0.7634	1.3322E-02	0.8326	2.7040E-15	**0.8358**	2.6702E-16
	10	0.8486	5.4287E-02	0.8522	6.0860E-03	0.8548	5.3772E-02	0.8155	1.3899E-01	0.8509	3.3859E-03	0.7914	4.3354E-02	0.8634	2.7040E-15	**0.8668**	2.6702E-16
Test 3	2	0.8622	8.9270E-16	0.8647	8.9270E-16	0.8690	8.8140E-16	0.8598	1.3359E-02	0.8632	8.5880E-16	0.8030	7.3450E-15	0.8706	9.0400E-16	**0.8791**	8.9270E-17
	5	0.9056	3.4380E-03	0.9079	7.1640E-03	0.9128	6.1542E-15	0.8972	1.0638E-01	0.9068	5.9964E-15	0.8435	2.0303E-02	0.9146	6.3120E-15	**0.9235**	6.2331E-16
	7	0.9430	4.1078E-03	0.9451	6.1499E-03	**0.9605**	4.7985E-03	0.9271	1.3876E-01	0.7875	1.6046E-03	0.8776	3.0148E-02	0.9512	2.7040E-15	**0.9605**	2.6702E-16
	10	0.9595	7.5158E-03	0.9616	7.9980E-03	0.9658	2.1347E-02	0.9407	1.4107E-01	0.9598	2.7161E-03	0.8935	6.6963E-02	0.9680	4.5040E-16	**0.9774**	4.4477E-16
Test 4	2	0.8112	3.5629E-15	0.8136	3.5629E-15	0.8175	3.5178E-15	0.8085	2.3115E-03	0.8121	3.4276E-15	0.7555	2.9315E-14	0.8191	3.6080E-16	**0.8271**	3.5629E-16
	5	0.8911	8.9270E-04	0.8935	2.2911E-03	**0.8986**	4.3914E-15	0.8873	4.8050E-03	0.8921	4.2788E-15	0.8299	3.6595E-14	0.8898	4.5040E-16	0.8981	4.4477E-16
	7	0.9300	3.1060E-03	0.9327	3.8932E-03	**0.9384**	2.1294E-03	0.9249	1.4124E-02	0.9307	6.8400E-04	0.9346	2.5424E-02	0.9242	4.5040E-16	0.9369	4.4477E-16
	10	0.9565	2.2116E-03	**0.9689**	1.2033E-03	0.9635	4.0404E-04	0.9363	1.0487E-01	0.9572	4.9704E-04	0.9608	1.2541E-02	0.9410	3.6080E-16	0.9635	3.5629E-16
Test 5	2	0.8272	1.7775E-15	**0.8437**	2.8788E-03	0.8337	1.7550E-15	0.8249	7.5590E-03	0.8282	1.7100E-15	0.8313	1.4625E-14	0.8202	1.8000E-16	**0.8437**	1.7775E-16
	5	0.8675	7.6877E-03	0.8711	1.7350E-02	0.8738	8.8140E-16	**0.8846**	4.9903E-02	0.8595	8.5880E-16	0.8718	6.7132E-02	0.8706	9.0400E-17	0.8840	8.9270E-18
	7	0.9028	8.6853E-03	**0.9262**	1.1923E-02	0.9094	4.2354E-03	**0.9262**	1.1791E-01	0.8943	2.5688E-15	0.9066	2.9324E-02	0.9162	2.7040E-16	0.9198	2.6702E-17
	10	0.9343	4.1061E-02	0.9381	7.3272E-03	0.9415	6.0495E-02	0.9064	1.3081E-01	0.9279	5.0914E-03	0.9405	4.3272E-02	0.9509	3.6080E-16	**0.9545**	3.5629E-17
Test 6	2	0.7390	2.6702E-15	0.7411	2.6702E-15	0.7447	4.0716E-04	0.7362	3.2997E-03	0.7326	2.5688E-15	0.7426	2.1970E-14	0.7506	2.7040E-16	**0.7534**	2.6702E-16
	5	0.7967	2.6702E-15	0.7990	6.3990E-04	**0.8029**	1.6302E-03	0.7927	7.8202E-02	0.7898	2.5688E-15	0.8006	2.1970E-14	0.8012	2.7040E-16	**0.8029**	2.6702E-16
	7	0.8400	1.8328E-03	**0.8467**	9.3718E-04	**0.8467**	2.2698E-03	0.8209	1.5379E-01	0.8325	4.7272E-04	0.8439	5.1285E-14	0.8429	6.3120E-16	0.8422	6.2331E-16
	10	0.8750	2.8145E-03	0.8777	4.5327E-03	0.8816	1.1310E-03	0.8424	1.5632E-01	0.8671	2.0444E-04	0.8791	1.5582E-02	**0.8884**	1.8000E-14	0.8772	1.7775E-16
Test 7	2	0.7724	3.5629E-15	0.7747	3.5629E-15	0.7785	3.5178E-15	0.7697	4.1979E-03	0.7657	3.4276E-15	0.7762	2.9315E-14	**0.7845**	3.6080E-14	0.7747	3.5629E-16
	5	0.8454	1.7775E-15	0.8479	1.1139E-04	0.8520	1.0608E-03	0.8386	9.5342E-02	0.8381	1.7100E-15	0.8496	1.4625E-14	0.8587	1.8000E-14	**0.8620**	1.7775E-16
	7	0.9010	1.0349E-03	0.9034	3.6429E-03	0.9079	4.9374E-03	0.8818	1.7289E-01	0.8931	2.5688E-15	0.8214	2.1970E-14	0.9150	2.7040E-14	**0.9186**	2.6702E-17
	10	0.9311	7.9695E-03	0.9328	7.4290E-03	0.9371	8.1838E-03	0.9067	1.4445E-01	0.9212	1.4715E-03	0.8474	4.5527E-02	0.9383	4.5040E-15	**0.9474**	4.4477E-17
Test 8	2	0.7725	1.7775E-15	0.7748	1.7775E-15	0.7786	1.7550E-15	0.7702	3.9268E-03	0.7659	1.7100E-15	0.7044	1.4625E-14	0.7801	1.8000E-15	**0.7877**	1.7775E-17
	5	0.8518	3.5629E-15	0.8543	3.5629E-15	0.8585	1.0140E-02	0.8430	1.6146E-01	0.8444	3.4276E-15	0.7766	2.9315E-14	**0.8685**	3.6080E-15	0.8601	3.5629E-17
	7	0.9092	7.6237E-02	0.9143	6.6739E-03	**0.9293**	7.3976E-03	0.8704	2.5744E-01	0.9037	4.4982E-03	0.8310	0.0000E+00	0.9203	0.0000E+00	**0.9293**	0.0000E+00
	10	0.9451	4.2539E-02	0.9493	2.4764E-03	0.9526	3.9179E-02	0.9051	1.4088E-01	0.9385	9.1512E-04	0.8630	1.2590E-02	0.9560	9.0400E-16	**0.9653**	8.9270E-18

**Table 8:** mSAR Otsu in terms of fitness values competing overall for algorithms.

		LFD		HHO		SCA		EO		GSA		AOA		SAR		mSAR	
Test Image	nTh	Mean	Std	Mean	Std	Mean	Std	Mean	Std	Mean	Std	Mean	Std	Mean	Std	Mean	Std
Test 1	2	1228.3380	1.3192E-15	2154.5265	1.3304E-15	2161.1633	1.2965E-15	1766.6759	1.4033E-03	2163.2565	1.2794E-15	2162.2380	9.2416E-02	2183.2334	1.1987E-15	**2347.1712**	1.1288E-15
	5	2267.7534	1.3192E-15	2336.1469	1.3304E-15	2344.7112	1.8691E-02	1766.6759	1.0255E-02	2345.5462	6.4323E-03	2325.5931	1.2796E-15	2368.7855	1.1987E-15	**2417.2129**	1.1288E-15
	7	2345.8176	9.1611E-04	2403.3510	3.9913E-15	2414.7995	5.5820E-03	1764.1485	2.2057E-02	**2684.2501**	5.3306E-04	2399.9273	8.1752E-04	2439.4421	3.5961E-15	2445.3025	3.3864E-15
	10	2433.4497	9.1611E-04	2429.4427	7.3173E-03	2443.2505	1.0120E-02	1762.3227	3.7421E-02	**2848.9400**	2.3490E-02	2439.9649	1.6741E-02	2466.6090	1.1987E-15	2468.1193	1.1288E-15
Test 2	2	2231.9467	1.3192E-15	2673.6262	1.3304E-15	2681.6200	1.2965E-15	2240.5229	6.1169E-04	2684.2501	1.2509E-14	2684.7420	1.2796E-15	**2709.0176**	1.1987E-15	2375.9160	1.1288E-15
	5	2256.1142	1.2716E-14	2838.4327	4.3978E-02	2847.5512	4.3215E-02	2245.7175	1.3421E-02	2849.5214	2.9887E-02	2844.4739	3.4229E-02	**2876.6441**	1.1987E-15	2413.1053	1.1288E-15
	7	2282.9820	5.7532E-05	2911.0919	5.6543E-05	2923.6189	3.9974E-02	2251.5354	2.4359E-02	2922.0748	2.1713E-04	2905.9256	1.1196E-01	2953.4878	4.7949E-15	**2969.9479**	4.5151E-15
	10	2389.8843	1.9055E-03	2955.3494	4.0652E-03	2966.8889	3.8174E-03	2266.9606	4.7496E-02	2921.9471	4.8687E-03	2969.5291	1.2121E-01	2997.2955	5.9936E-15	**2998.4400**	5.6439E-15
Test 3	2	1349.9321	1.3192E-15	1700.6816	1.3304E-15	1707.9356	1.2965E-15	1346.5997	3.0009E-04	1709.6107	3.8025E-14	1700.1406	1.2796E-15	1725.3852	1.1987E-15	**1809.8735**	1.1288E-15
	5	1436.6207	1.5647E-02	1800.7810	8.4999E-03	1808.0657	4.8257E-05	1368.6302	2.0869E-03	**1869.1703**	1.4784E-02	1793.0477	6.3980E-15	1826.5383	1.3818E-02	**1869.1703**	1.3012E-02
	7	1855.0663	1.3558E-03	1819.5871	7.3173E-02	1866.9733	1.6926E-03	1816.7927	3.3211E-02	1865.1461	6.8588E-02	1849.2056	5.2250E-02	**1886.3811**	2.4008E-15	1865.5869	2.2607E-15
	10	1883.8377	3.8843E-03	1883.7849	7.1695E-02	1895.8753	3.2772E-03	1855.9531	6.5486E-02	1864.1770	9.7728E-02	1885.1025	1.1943E-01	1915.1461	3.5961E-15	**1917.1353**	3.3864E-15
Test 4	2	3342.3522	5.2768E-15	4005.5150	5.3217E-15	4017.6015	1.1308E-06	3378.4302	9.3552E-04	4021.2621	5.1174E-15	4019.5170	5.1184E-15	4058.6485	4.7949E-15	**4104.8147**	4.9869E-15
	5	3357.6885	1.2313E-05	4087.9590	3.9913E-15	4100.7145	4.3215E-04	3532.9058	4.3897E-03	4104.5360	3.8381E-15	4104.5710	3.8388E-15	4142.5629	3.5961E-15	**4165.3647**	3.7402E-15
	7	3531.2658	1.2826E-04	4146.2650	1.8145E-04	4161.2052	1.5702E-04	3588.5685	1.2270E-02	4160.5588	5.3306E-04	4157.2434	6.6113E-05	**4203.7193**	1.1987E-15	4197.6941	1.2467E-15
	10	3566.7480	1.3962E-02	4180.4044	2.2913E-02	4195.6883	5.2939E-03	3628.7443	2.4755E-02	4192.1343	2.9247E-02	4221.9163	2.6943E-02	**4229.6436**	1.1987E-15	4221.8588	1.2467E-15
Test 5	2	2022.1883	1.3192E-15	2137.3286	1.3304E-15	2144.5158	1.2965E-15	2174.5007	3.1880E-04	2146.5530	1.0093E-14	2144.6728	1.2796E-15	2166.4247	1.1987E-15	**2230.4867**	1.2467E-15
	5	2105.3476	2.6970E-14	2220.8704	3.9913E-15	2228.2801	1.0120E-02	2205.3313	6.0449E-03	2229.1199	4.6199E-14	2216.7077	3.8388E-15	2251.0460	3.5961E-15	**2280.5788**	3.7402E-15
	7	2116.4290	7.6953E-04	**2310.5996**	9.9412E-02	2277.8903	5.9781E-02	2268.2821	6.0809E-02	2230.2398	7.2852E-02	2277.3147	9.5970E-02	2301.6064	5.9936E-15	2309.2243	6.1459E-15
	10	2205.3323	1.0370E-02	2295.5602	9.7934E-02	2306.9782	4.9698E-02	2311.4290	8.3837E-02	2275.9562	1.0128E-01	2257.8829	1.1090E-01	**2338.5126**	3.5961E-05	2325.0029	1.2292E-15
Test 6	2	2362.7631	2.6421E-15	2779.8693	2.6645E-15	2788.2653	2.5965E-15	2462.6670	5.3972E-04	2790.9999	2.5623E-15	2791.1473	2.5628E-15	2816.7524	2.4008E-15	**2899.2436**	2.4618E-15
	5	2543.9257	6.2295E-04	2986.4010	4.8782E-05	**3026.3672**	1.0444E-03	2696.2576	4.3178E-03	2999.7423	9.2398E-04	2969.9597	1.6919E-05	**3026.3672**	1.0988E-03	3010.8257	1.2292E-03
	7	2657.2510	2.3123E-04	3092.6749	2.6424E-02	3107.7510	3.2196E-04	2891.4927	2.5367E-02	3109.2573	2.5658E-02	3110.3556	4.4786E-02	3138.7965	5.9936E-15	**3167.6023**	6.1494E-15
	10	2673.9420	2.1620E-03	3150.4793	1.9587E-03	3164.3702	5.5460E-03	2919.2860	3.9580E-02	3115.0013	5.1885E-03	3166.8879	1.1445E-02	3196.6999	0.0000E+00	**3201.8355**	0.0000E+00
Test 7	2	1824.5625	1.3192E-15	**1938.2958**	8.7956E-05	1790.9441	5.2579E-05	1769.3905	1.2594E-03	1792.4297	7.5695E-05	1792.0819	4.0165E-05	1809.2418	1.1987E-15	**1938.2958**	1.2804E-15
	5	1835.9877	3.9576E-15	1928.5638	2.8752E-04	1936.3773	7.9228E-04	1917.0802	5.3253E-03	1935.5520	5.6860E-04	1939.3067	3.8388E-15	1956.1609	3.5961E-15	**2014.0182**	3.8412E-15
	7	1994.1837	3.6644E-04	1994.5436	2.8087E-03	**2061.9455**	2.8450E-03	1983.5858	1.5148E-02	2009.5965	2.2744E-03	2012.5464	1.8483E-03	2031.9621	8.3910E-15	**2061.9455**	8.9629E-15
	10	2002.4547	2.0521E-03	2045.0262	2.6608E-03	2089.0842	5.8701E-03	2033.1707	3.1772E-02	2008.9027	4.3000E-03	2053.9605	5.1539E-03	2080.4898	9.5897E-15	**2091.3972**	9.2998E-15
Test 8	2	3404.3880	5.2768E-15	**3424.3880**	5.3217E-15	3370.3381	3.7093E-05	2754.5731	4.6776E-03	3373.5875	5.1174E-15	3373.5524	1.5249E-01	3404.7155	4.7949E-15	**3424.3880**	4.6499E-15
	5	3447.8143	2.6970E-04	3520.6757	3.9913E-15	3534.2506	6.3383E-02	2918.6109	2.7814E-02	3536.3315	1.1017E-03	3537.8986	3.8388E-15	3570.3593	3.5961E-15	**3599.4514**	3.4874E-15
	7	3640.4737	6.9624E-04	3579.3755	1.2528E-04	3596.1054	1.8151E-02	2945.7964	4.3178E-02	3597.8396	1.0306E-03	3599.7578	6.3980E-04	3632.5749	3.5961E-15	**3661.2643**	3.4874E-15
	10	3557.6969	9.6008E-03	**3692.4467**	7.2434E-03	3638.5602	1.2749E-02	2963.2611	2.3892E-01	3626.7722	8.5646E-03	3618.3636	7.3577E-02	3675.7346	2.4008E-15	**3692.4467**	2.3282E-15

**Table 9:** mSAR Otsu in terms of PSNR values competed overall for algorithms.

		LFD		HHO		SCA		EO		GSA		AOA		SAR		mSAR	
Test Image	nTh	Mean	Std	Mean	Std	Mean	Std	Mean	Std	Mean	Std	Mean	Std	Mean	Std	Mean	Std
Test 1	2	18.0774	5.2812E-14	18.0789	5.2380E-14	18.0789	5.2488E-14	18.0266	2.8130E-01	18.0600	5.2272E-14	18.0617	5.2380E-14	**21.2302**	5.2596E-14	19.0774	5.2488E-14
	5	21.2304	4.1076E-14	21.2322	7.0810E-02	21.2322	6.1722E-02	20.9442	7.5175E-01	21.2100	0.0000E+00	21.2120	0.0000E+00	22.1965	0.0000E+00	**22.2304**	0.0000E+00
	7	22.5967	1.9022E-14	22.5987	1.9061E-01	22.5987	2.0169E-01	22.0904	2.6675E+00	22.5750	3.4896E-14	22.5772	7.4205E-02	24.4883	3.5113E-14	**24.5967**	3.5041E-14
	10	24.4885	1.8778E-14	24.4906	9.9910E-02	24.4906	1.4677E-01	23.1324	3.5987E+00	24.4650	7.4052E-16	24.4673	1.0428E-01	24.1854	7.0128E-14	**24.9885**	6.9984E-14
Test 2	2	16.1856	3.5257E-14	16.1869	3.4969E-14	16.1869	3.5041E-14	16.0468	1.2028E-01	16.1700	3.4896E-14	16.1715	3.4969E-14	**18.2874**	3.5113E-14	16.1856	3.5041E-14
	5	18.2876	5.2812E-14	18.2891	8.0025E-02	18.2891	5.2488E-14	18.1308	4.9955E-01	18.2700	5.2272E-14	18.2717	5.2380E-14	**19.7588**	5.2596E-14	18.2876	5.2488E-14
	7	19.7590	1.9560E-16	19.7607	2.8615E-02	19.6556	1.1518E+00	19.0686	3.1186E+00	19.7400	0.0000E+00	19.7419	1.2125E-02	**20.3894**	0.0000E+00	19.7590	0.0000E+00
	10	20.4947	1.3839E-16	20.6016	1.5617E+00	20.3913	1.0012E+00	19.6938	2.8712E+00	20.4750	1.2826E+00	**20.7920**	1.7703E+00	20.1854	7.0128E-14	20.3896	6.9984E-14
Test 3	2	16.1856	6.1614E-14	**18.6027**	6.1110E-14	16.1869	6.1236E-14	16.0468	2.2456E-16	16.1700	6.0984E-14	16.1715	6.1110E-14	**18.6027**	6.1362E-14	16.1856	6.1236E-14
	5	18.6029	1.8093E-02	18.6045	3.0798E-01	18.6045	3.5041E-14	**19.9350**	3.8121E+00	18.5850	3.4896E-14	18.5868	1.3095E-02	19.2302	3.5113E-14	18.6029	3.5041E-14
	7	21.3355	1.7702E-01	21.2322	2.4347E-01	**22.7616**	2.0169E-01	20.2148	5.5775E+00	21.2100	7.1632E-16	21.2120	1.5569E-16	22.7016	5.2596E-14	21.2304	5.2488E-11
	10	22.8069	3.6920E-01	22.9140	9.6030E-01	22.7038	1.1713E+00	21.3610	6.1110E+00	22.6800	2.4200E-16	22.7872	4.8985E-01	22.8129	3.5113E-14	**22.8918**	3.5041E-14
Test 4	2	18.8131	0.0000E+00	**21.3353**	0.0000E+00	18.8147	0.0000E+00	18.6518	5.7715E-02	18.7950	0.0000E+00	18.7968	0.0000E+00	**21.3353**	0.0000E+00	18.8131	0.0000E+00
	5	21.3355	1.7702E-16	21.3373	4.4135E-02	21.3373	5.2488E-14	21.1526	3.5696E-01	21.3150	5.2272E-14	21.3170	5.2380E-14	22.3322	5.2596E-14	**22.6355**	5.2488E-13
	7	23.3324	6.3081E-02	23.3344	1.0573E-01	23.3344	3.8880E-02	23.0282	5.4805E-01	23.3100	2.4200E-16	23.3122	6.4020E-02	23.9087	3.5113E-14	**23.4324**	3.5041E-14
	10	24.9089	1.2959E-01	24.9111	8.4875E-02	**24.9689**	7.9218E-02	23.7576	3.4920E+00	24.8850	1.7424E-02	24.8874	6.4020E-16	24.7650	0.0000E+00	**24.9689**	0.0000E+00
Test 5	2	15.7652	6.1614E-14	15.7665	6.0140E-02	15.7665	6.1236E-14	15.6300	1.9982E-01	15.7500	6.0984E-14	15.7515	6.1110E-14	15.7588	6.1362E-14	**16.7652**	6.1236E-13
	5	19.7590	1.2616E-16	**19.7607**	2.9973E-01	**19.7607**	6.9984E-14	19.5896	1.4647E+00	19.7400	6.9696E-14	19.7419	1.3289E-01	19.7557	7.0128E-14	19.7590	6.9984E-14
	7	21.8610	1.6871E-16	21.8629	2.7063E-01	21.7578	9.3798E-02	21.1526	4.2923E+00	21.7350	5.2272E-14	21.7371	4.8500E-02	21.3832	5.2596E-14	**22.7559**	5.2488E-13
	10	24.2783	1.7653E+00	24.2804	1.8527E-01	24.1753	2.7070E+00	22.2988	4.8064E+00	24.3600	1.1132E-01	24.2573	1.4841E-01	24.1313	5.2596E-14	**24.5834**	5.2488E-14
Test 6	2	17.1315	5.2812E-14	**17.1329**	5.2380E-14	**17.1329**	6.9498E-04	16.9846	8.1480E-02	17.1150	5.2272E-14	17.1166	5.2380E-14	17.3384	5.2596E-14	17.1315	5.2488E-14
	5	19.3386	7.0416E-14	19.3402	2.2795E-02	19.3402	2.6341E-02	19.0686	1.6733E+00	19.3200	6.9696E-14	19.3218	6.9840E-14	**19.7557**	7.0128E-14	19.3386	6.9984E-14
	7	21.7559	4.8802E-16	**21.7578**	4.4620E-02	**21.7578**	1.2101E-01	20.7358	4.9955E+00	21.7350	1.3068E-02	21.7371	5.2380E-14	21.4373	5.2596E-14	21.7559	5.2488E-12
	10	23.4375	1.0269E-01	**23.4395**	8.2935E-02	**23.4395**	6.2694E-02	21.7778	4.3893E-16	23.4150	7.7440E-03	23.4172	5.4320E-02	23.8160	8.7660E-14	23.4375	8.7480E-14
Test 7	2	16.8162	2.6455E-14	16.8176	2.6239E-14	16.8176	2.6293E-14	16.6720	1.0234E-01	16.8000	2.6184E-14	16.8016	2.6239E-14	16.1282	2.6347E-14	**17.8162**	2.6293E-14
	5	19.1284	3.5257E-14	19.1300	4.6075E-02	19.1300	1.0595E-01	18.8602	1.8236E+00	19.1100	3.4896E-14	19.1118	3.4969E-14	19.7557	3.5113E-14	**20.1284**	3.5041E-14
	7	21.7559	9.3399E-16	21.7578	5.4320E-02	21.7578	1.4337E-01	20.8400	4.9470E+00	21.7350	8.7120E-14	21.7371	8.7300E-14	21.3322	8.7660E-14	**21.9859**	8.7480E-14
	10	23.3324	1.6528E-01	23.3344	1.2804E-01	23.3344	1.5260E-01	22.0904	4.2195E+00	23.3100	3.7268E-16	23.3122	2.5705E-02	**23.4024**	7.0128E-14	**23.4024**	6.9984E-14
Test 8	2	15.3447	0.0000E+00	15.3461	0.0000E+00	15.3461	0.0000E+00	15.2132	1.2125E-01	15.3300	0.0000E+00	15.3315	0.0000E+00	**16.1792**	0.0000E+00	15.3447	0.0000E+00
	5	20.1794	1.7604E-14	20.1811	1.7460E-14	20.1811	6.3180E-01	19.5896	6.0625E+00	20.1600	1.7424E-14	20.1619	1.7460E-14	20.2812	1.7532E-14	**20.4794**	1.7496E-14
	7	22.0712	3.7164E+00	22.1782	3.5114E-01	22.1782	6.8526E-01	19.1728	1.1786E+01	22.2600	2.4636E-01	22.2621	3.4969E-14	23.5424	3.5113E-14	**23.6814**	3.5041E-14
	10	23.3324	8.8020E-01	**23.5826**	1.2174E-01	23.4395	1.8760E+00	21.8820	7.5175E+00	23.5200	1.1084E-01	23.5222	1.6830E-01	23.3401	5.2596E-14	**23.5826**	5.2488E-14

**Table 10:** mSAR Otsu in terms of SSIM values overall competed for algorithms.

		LFD		HHO		SCA		EO		GSA		AOA		SAR		mSAR	
Test Image	nTh	Mean	Std	Mean	Std	Mean	Std	Mean	Std	Mean	Std	Mean	Std	Mean	Std	Mean	Std
Test 1	2	0.8003	2.4625E-14	0.8010	1.9447E-15	0.8007	1.9628E-15	0.8009	6.2930E-03	0.8008	2.0079E-15	0.8008	1.9853E-15	**0.8011**	1.9575E-15	0.8010	1.9614E-15
	5	0.8409	3.1231E-03	0.8417	3.5876E-03	0.8414	3.7514E-04	0.8405	3.2613E-02	0.8415	3.5126E-15	0.8415	3.4732E-15	0.8418	3.4245E-15	**0.8419**	3.4314E-15
	7	0.8659	2.5061E-02	0.8667	2.3759E-03	0.8664	1.7060E-03	0.8624	2.4832E-02	0.8665	2.0079E-15	0.8665	9.8165E-04	0.8668	1.9575E-15	**0.8672**	1.9614E-15
	10	0.8940	5.1433E-02	0.8949	3.7514E-03	0.8946	2.3066E-03	0.8822	3.7970E-02	0.8946	9.0821E-03	0.8946	1.5891E-03	**0.8950**	1.4670E-15	**0.8950**	1.4700E-15
Test 2	2	0.6731	6.1698E-15	0.6738	4.8726E-16	0.6735	4.9178E-16	**0.6747**	8.8867E-03	0.6736	5.0308E-16	0.6736	4.9743E-16	0.6738	4.9045E-16	0.6738	4.9144E-16
	5	0.7523	2.4625E-14	0.7530	5.2606E-03	0.7528	1.9628E-15	0.7519	2.8361E-02	0.7528	2.0079E-15	0.7528	1.9853E-15	0.7531	1.9575E-15	**0.7535**	1.9614E-15
	7	0.7971	7.7532E-03	**0.7980**	7.5460E-04	0.7955	2.7635E-02	0.7842	9.0142E-02	0.7977	2.0079E-15	0.7977	4.4900E-04	0.7980	1.9575E-15	**0.7980**	1.9614E-15
	10	0.8221	5.1433E-01	0.8271	4.7001E-02	0.8205	3.9951E-02	0.8040	8.2489E-02	0.8237	3.9489E-02	0.8331	5.3704E-02	0.8199	9.7657E-16	**0.8208**	9.7853E-16
Test 3	2	0.8221	6.1698E-15	**0.8235**	4.8726E-16	0.8226	4.9178E-16	0.8228	1.0077E-02	0.8227	5.0308E-16	0.8227	4.9743E-16	0.8230	4.9045E-16	**0.8235**	4.9144E-16
	5	0.8899	2.2932E-03	**0.8918**	6.7698E-03	0.8904	3.4337E-15	0.8843	9.2268E-02	0.8905	3.5126E-15	0.8905	1.3250E-04	0.8908	3.4245E-15	0.8908	3.4314E-15
	7	**0.9524**	4.4663E-02	0.9523	4.9588E-03	0.9519	4.0735E-03	0.9323	1.1353E-01	0.9509	1.4647E-03	0.9520	3.2223E-03	0.9513	1.4670E-15	0.9513	1.4700E-15
	10	0.9732	6.4974E-02	**0.9752**	1.0909E-02	0.9727	1.5145E-02	0.9552	1.0502E-01	0.9728	3.0452E-04	0.9739	6.9552E-03	0.9732	2.4436E-15	0.9732	2.4485E-15
Test 4	2	0.7898	1.2285E-14	0.7906	9.7020E-16	0.7903	9.7920E-16	0.7904	3.7460E-03	0.7904	1.0017E-15	0.7904	9.9045E-16	**0.7907**	9.7657E-16	**0.7907**	9.7853E-16
	5	0.8826	2.1185E-02	0.8834	4.5707E-03	0.8831	2.4502E-15	0.8833	1.2543E-02	0.8832	2.5065E-15	0.8832	2.4783E-15	0.8832	2.4436E-15	**0.8835**	2.4485E-15
	7	0.9253	1.0483E-01	0.9272	1.3885E-02	0.9237	2.8636E-03	**0.9291**	3.0572E-02	0.9238	1.6517E-03	0.9270	1.2370E-02	0.9240	1.4670E-15	0.9242	1.4700E-15
	10	0.9607	2.1076E-02	0.9616	1.8240E-03	0.9613	9.9661E-04	0.9437	6.8457E-02	0.9614	2.9962E-04	0.9614	5.7226E-04	0.9616	2.4436E-15	**0.9619**	2.4485E-15
Test 5	2	0.8742	1.2285E-14	0.8751	1.8197E-03	0.8747	9.7920E-16	**0.8760**	6.6756E-03	0.8748	1.0017E-15	0.8748	9.9045E-16	0.8750	9.7657E-16	0.8752	9.7853E-16
	5	0.9274	9.0636E-02	**0.9304**	1.5998E-02	0.9269	9.7920E-16	0.9270	5.0174E-02	0.9270	1.0017E-15	0.9280	7.7475E-03	0.9271	9.7657E-16	0.9273	9.7853E-16
	7	0.9493	4.5482E-02	**0.9502**	5.3900E-03	0.9488	2.3718E-03	0.9458	5.1024E-02	0.9489	2.0079E-15	0.9489	1.2370E-03	0.9490	1.9575E-15	**0.9502**	1.9614E-15
	10	0.9836	2.7791E-01	0.9856	3.0874E-03	0.9842	3.5208E-02	0.9615	6.5906E-02	0.9864	2.1904E-03	0.9864	2.4387E-03	0.9866	4.9045E-16	**0.9869**	4.9144E-16
Test 6	2	0.7909	1.8455E-14	**0.7919**	1.4575E-15	0.7913	1.2229E-03	0.7915	5.9103E-03	0.7914	1.5048E-15	0.7914	1.4879E-15	0.7916	1.4670E-15	**0.7919**	1.4700E-15
	5	0.8326	0.0000E+00	**0.8334**	1.1039E-04	0.8330	2.5024E-03	0.8322	3.0062E-02	0.8331	0.0000E+00	0.8331	0.0000E+00	0.8333	0.0000E+00	**0.8334**	0.0000E+00
	7	0.8972	6.8796E-03	0.8980	3.1521E-04	0.8977	1.7452E-03	0.8739	1.1991E-01	0.8978	8.0581E-05	0.8978	0.0000E+00	0.8979	0.0000E+00	**0.8986**	0.0000E+00
	10	0.9211	2.1239E-02	0.9220	2.3630E-03	0.9217	6.8762E-04	0.9010	8.1213E-02	0.9217	3.7530E-04	0.9217	1.0213E-03	0.9219	2.4436E-15	**0.9225**	2.4485E-15
Test 7	2	0.7617	0.0000E+00	0.7624	0.0000E+00	0.7621	0.0000E+00	0.7623	5.0174E-03	0.7622	0.0000E+00	0.7622	0.0000E+00	**0.7626**	0.0000E+00	**0.7626**	0.0000E+00
	5	0.8419	1.2285E-14	**0.8428**	8.5378E-04	0.8414	1.8627E-03	0.8374	5.6126E-02	0.8425	1.0017E-15	0.8425	9.9045E-16	0.8427	9.7657E-16	0.8427	9.7853E-16
	7	0.9232	5.0942E-02	0.9241	4.0662E-03	0.9237	6.9632E-03	0.9031	1.3904E-01	0.9238	2.5065E-15	0.9238	2.4783E-15	0.9235	2.4436E-15	**0.9243**	2.4485E-15
	10	**0.9597**	7.9170E-02	0.9585	6.3818E-03	0.9581	6.3539E-03	0.9364	9.9072E-02	0.9572	2.4531E-04	0.9572	4.2347E-03	0.9568	0.0000E+00	0.9575	0.0000E+00
Test 8	2	0.7304	2.4625E-14	0.7310	1.9447E-15	0.7309	1.9628E-15	0.7300	5.1874E-03	0.7309	2.0079E-15	0.7309	1.9853E-15	0.7307	1.9575E-15	**0.7318**	1.9614E-15
	5	0.9201	1.2285E-14	**0.9210**	9.7020E-16	0.9206	1.2055E-02	0.9062	2.0197E-01	0.9207	1.0017E-15	0.9207	9.9045E-16	0.9204	9.7657E-16	**0.9210**	9.7853E-16
	7	0.9566	1.1630E+00	0.9616	2.9839E-03	0.9613	8.7910E-03	0.8791	3.1167E-01	0.9624	1.8921E-03	0.9624	4.9743E-16	0.9620	4.9045E-16	**0.9627**	4.9144E-16
	10	0.9795	1.4360E-01	**0.9826**	1.6213E-03	0.9800	2.0846E-02	0.9521	1.7051E-01	0.9822	1.0907E-03	0.9822	1.6948E-03	0.9818	2.4436E-15	0.9825	2.4485E-15

**Table 11:** mSAR Otsu in terms of FSIM values overall competed for algorithms.

		LFD		HHO		SCA		EO		GSA		AOA		SAR		mSAR	
Test Image	nTh	Mean	Std	Mean	Std	Mean	Std	Mean	Std	Mean	Std	Mean	Std	Mean	Std	Mean	Std
Test 1	2	0.8005	1.9063E-15	0.8005	1.8725E-15	0.8005	1.8759E-15	**0.8006**	1.0805E-02	0.7997	1.9401E-15	0.7982	1.9131E-15	0.8005	1.8725E-15	0.8002	1.8759E-15
	5	0.8457	8.9112E-05	0.8457	1.6354E-03	0.8457	1.3209E-04	**0.8458**	1.2488E-15	0.8449	1.2915E-15	0.8433	1.2735E-15	0.8457	1.2465E-15	0.8418	1.2488E-15
	7	0.8789	5.1117E-03	0.8794	3.6486E-03	0.8795	4.2458E-03	**0.8800**	3.1247E-15	0.8790	3.2316E-15	0.8773	1.8343E-03	0.8799	3.1190E-15	0.8691	3.1247E-15
	10	0.9199	3.1590E-03	**0.9202**	1.6471E-03	0.9202	2.5890E-03	**0.9202**	9.6348E-15	0.9188	1.4372E-02	0.9173	1.5747E-03	0.9200	4.3711E-15	0.9201	4.3790E-15
Test 2	2	0.7249	1.9063E-15	0.7249	1.8725E-15	0.7249	1.8759E-15	0.7232	8.1872E-03	0.7243	1.9401E-15	0.7229	1.9131E-15	0.7249	1.8725E-15	**0.7250**	1.8759E-15
	5	0.7823	1.2690E-15	**0.7825**	4.2585E-03	0.7823	1.2488E-15	0.7791	2.8914E-02	0.7815	1.2915E-15	0.7800	1.2735E-15	0.7823	1.2465E-15	0.7824	1.2488E-15
	7	0.8340	1.6652E-03	0.8339	2.5451E-03	0.8313	6.0495E-02	0.8343	1.2718E-01	0.8334	1.9401E-15	0.8317	1.1600E-03	**0.8346**	1.8725E-15	0.8341	1.8759E-15
	10	0.8636	3.8757E-02	0.8647	4.2679E-03	0.8631	3.8261E-02	**0.8652**	9.8151E-02	0.8640	2.5572E-03	0.8622	3.7751E-03	0.8651	1.8725E-15	**0.8652**	1.8759E-15
Test 3	2	0.8774	6.3732E-16	0.8774	6.2602E-16	0.8774	6.2715E-16	**0.8775**	9.4338E-03	0.8766	6.4862E-16	0.8749	6.3958E-16	0.8774	6.2602E-16	0.8773	6.2715E-16
	5	0.9216	2.4545E-03	0.9212	5.0238E-03	**0.9219**	4.3790E-15	0.9218	7.5124E-15	0.9208	4.5289E-15	0.9190	1.7679E-03	0.9217	4.3711E-15	0.9216	4.3790E-15
	7	**0.9596**	2.9326E-03	0.9589	4.3127E-03	0.9589	3.4143E-03	0.9587	9.7989E-15	0.9578	1.2119E-03	0.9561	2.6252E-03	0.9586	1.8725E-15	0.9583	1.8759E-15
	10	**0.9764**	5.3657E-03	0.9757	5.6087E-03	0.9752	1.5189E-02	0.9756	9.9618E-15	0.9746	2.0514E-03	0.9734	5.8309E-03	0.9755	3.1190E-15	0.9750	3.1247E-15
Test 4	2	0.8255	2.5436E-15	0.8255	2.4985E-15	0.8255	2.5031E-15	**0.8256**	1.6323E-11	0.8247	2.5887E-15	0.8231	2.5527E-15	0.8255	2.4985E-15	**0.8256**	2.5031E-15
	5	0.9068	6.3732E-04	0.9066	1.6067E-03	0.9068	3.1247E-15	**0.9069**	3.3932E-03	0.9059	3.2316E-15	0.9042	3.1866E-15	0.9068	3.1190E-15	0.9065	3.1247E-15
	7	**0.9464**	2.2175E-03	**0.9464**	2.7302E-03	0.9460	1.5152E-03	0.9461	9.9742E-03	0.9451	5.1660E-04	0.9437	2.2139E-03	0.9460	3.1190E-15	0.9459	3.1247E-15
	10	**0.9733**	1.5789E-03	0.9730	8.4385E-04	0.9729	2.8749E-04	0.9730	7.4054E-02	0.9720	3.7540E-04	0.9720	1.0920E-03	0.9729	2.4985E-15	0.9724	2.5031E-15
Test 5	2	0.8418	1.2690E-15	**0.8419**	2.0188E-03	0.8418	1.2488E-15	**0.8419**	5.3380E-06	0.8410	1.2915E-15	0.8410	1.2735E-15	0.8418	1.2465E-15	**0.8419**	1.2488E-15
	5	0.8828	5.4885E-03	**0.8839**	1.2167E-02	0.8823	6.2715E-16	**0.8839**	3.5241E-02	0.8815	6.4862E-16	0.8820	5.8456E-03	0.8823	6.2602E-16	0.8824	6.2715E-16
	7	0.9187	6.2006E-03	0.9192	8.3615E-03	0.9182	3.0137E-03	**0.9195**	8.3266E-02	0.9171	1.9401E-15	0.9172	2.5535E-03	0.9180	1.8725E-15	0.9181	1.8759E-15
	10	0.9508	2.9314E-02	0.9519	5.1383E-03	0.9507	4.3045E-02	**0.9528**	9.2372E-02	0.9515	3.8453E-03	0.9515	3.7680E-03	**0.9528**	2.4985E-15	0.9523	2.5031E-15
Test 6	2	0.7520	1.9063E-15	0.7520	1.8725E-15	0.7520	2.8971E-04	0.7489	2.3302E-03	0.7513	1.9401E-15	0.7513	1.9131E-15	0.7520	1.8725E-15	**0.7521**	1.8759E-15
	5	0.8108	1.9063E-15	0.8107	4.4874E-04	0.8107	1.1600E-03	0.8063	5.5225E-02	0.8100	1.9401E-15	0.8100	1.9131E-15	**0.8109**	1.8725E-15	**0.8109**	1.8759E-15
	7	0.8548	1.3085E-03	0.8546	6.5721E-04	**0.8549**	1.6151E-03	0.8350	1.0860E-01	0.8538	3.5703E-04	0.8538	4.4657E-15	0.8546	4.3711E-15	0.8547	4.3790E-15
	10	0.8905	2.0094E-03	0.8906	3.1786E-03	0.8902	8.0475E-04	0.8569	1.1039E-01	0.8892	1.5441E-04	0.8894	1.3569E-03	0.8901	1.2465E-15	**0.8909**	1.2488E-15
Test 7	2	0.7860	2.5436E-15	0.7860	2.4985E-15	0.7860	2.5031E-15	0.7829	2.9645E-03	0.7853	2.5887E-15	0.7853	2.5527E-15	0.7860	2.4985E-15	**0.7861**	2.5031E-15
	5	0.8603	1.2690E-15	0.8603	7.8114E-05	0.8603	7.5480E-04	0.8530	6.7329E-02	0.8595	1.2915E-15	0.8603	1.2735E-15	0.8603	1.2465E-15	**0.8604**	1.2488E-15
	7	**0.9169**	7.3884E-04	0.9167	2.5547E-03	0.9167	3.5132E-03	0.8970	1.2209E-01	0.9159	1.9401E-15	0.9168	1.9131E-15	0.9168	1.8725E-15	**0.9169**	1.8759E-15
	10	0.9476	5.6896E-03	0.9464	5.2097E-03	0.9462	5.8231E-03	0.9223	1.0201E-01	0.9447	1.1114E-03	0.9458	3.9644E-03	0.9456	3.1190E-15	**0.9479**	3.1247E-15
Test 8	2	0.7862	1.2690E-15	0.7862	1.2465E-15	0.7862	1.2488E-15	**0.7864**	2.7730E-13	0.7854	1.2915E-15	0.7862	1.2735E-15	**0.7864**	1.2465E-15	0.7862	1.2488E-15
	5	0.8668	2.5436E-15	0.8668	2.4985E-15	**0.8669**	7.2150E-03	**0.8669**	1.1402E-08	0.8660	2.5887E-15	0.8668	2.5527E-15	0.8668	2.4985E-15	**0.8669**	2.5031E-15
	7	0.9252	5.4428E-02	**0.9278**	4.6802E-03	0.9270	5.2637E-03	0.9276	1.8180E-07	0.9267	3.3973E-03	0.9275	0.0000E+00	0.9275	0.0000E+00	0.9276	0.0000E+00
	10	0.9618	3.0370E-02	0.9632	1.7366E-03	0.9618	2.7877E-02	**0.9635**	9.9484E-08	0.9625	6.9115E-04	0.9633	1.0963E-03	0.9634	6.2602E-16	**0.9635**	6.2715E-16

## Data Availability

All data generated or analysed during this study are included in this published article [54].

## Appendix A. The Results of Optimal Threshold Values and the Wilcoxon Signed-Rank Test

The results of optimal threshold values and the Wilcoxon signed-rank over the set of test images test obtained by the mSAR algorithm and other competitive algorithms using Fuzzy Entropy and the Otsu method are as follows:

**Table A1:** The optimal threshold values are obtained by the Fuzzy Entropy method.

		mSAR			SAR		
Test Image	nTh	Red	Green	Blue	Red	Green	Blue
Test 1	2	68 115	99 175	70 100	70 114	98 176	73 95
	5	15 43 86 89 137	63 69 113 172 198	46 84 98 138 150	16 39 86 92 143	60 66 110 176 200	48 81 100 143 149
	7	53 97 113 149 189 196 210	76 103 118 134 165 195 213	60 107 109 112 139 209 246	50 85 120 150 189 200 210	70 109 112 130 156 199 230	56 110 114 116 125 201 238
	10	23 68 87 160 171 185 189 200 216 218	38 47 56 72 96 122 156 219 222 230	49 82 100 183 198 203 221 233 235 247	68 77 123 134 135 160 177 184 200 246	80 89 96 129 160 159 179 190 197 220	87 102 103 110 136 151 216 236 246 253
Test 2	2	93 107	40 44	100 205	92 108	41 42	99 205
	5	91 136 151 170 198	50 51 129 178 239	99 154 168 191 215	85 130 150 166 202	45 63 125 178 237	92 140 162 186 211
	7	23 47 74 108 134 168 196	34 54 62 70 128 161 177	75 85 119 141 157 210 235	20 44 68 112 139 155 201	32 51 54 64 117 160 169	72 78 114 137 164 199 221
	10	29 63 82 94 95 123 132 201 203 216	28 41 70 81 91 98 98 105 194 230	50 66 104 107 125 127 149 149 183 241	30 63 75 98 99 123 142 200 210 220	25 34 66 88 91 100 120 132 178 226	46 67 104 107 132 146 166 172 185 232
Test 3	2	18 166	77 130	100 210	20 165	76 132	102 208
	5	46 55 167 178 191	19 42 48 116 169	61 69 82 98 200	44 56 170 177 195	20 38 41 112 172	73 79 89 106 209
	7	16 35 74 109 165 178 189	83 113 127 160 181 185 190	96 130 139 159 215 236 239	18 35 76 112 150 177 190	82 113 125 156 182 184 192	98 136 146 160 211 213 240
	10	21 39 56 56 68 79 86 95 119 201	43 60 63 70 72 99 122 125 234 253	64 119 149 170 185 186 188 219 221 254	20 35 55 57 65 80 84 102 120 211	36 56 61 72 76 100 123 134 221 250	62 112 140 166 176 177 190 213 226 239
Test 4	2	23 98	69 112	35 105	23 96	70 111	31 107
	5	32 36 97 119 181	73 163 170 227 243	138 174 193 196 206	33 36 100 121 175	69 162 168 221 244	135 180 193 201 209
	7	35 43 81 149 171 217 241	40 49 82 125 129 131 178	39 53 59 155 159 183 244	32 46 78 149 168 214 236	35 52 87 123 138 142 160	28 46 63 142 155 178 235
	10	17 23 24 27 28 71 93 122 156 201	56 107 107 142 163 203 208 217 219 241	61 68 93 115 155 188 196 197 223 244	14 20 24 27 30 76 92 122 155 216	56 103 116 142 166 209 208 218 220 241	65 73 93 111 145 188 193 200 223 240
Test 5	2	149 222	130 186	44 208	149 220	133 187	46 207
	5	29 33 59 186 241	26 26 75 116 186	54 128 165 226 241	30 36 62 186 234	26 36 77 111 166	52 122 163 224 239
	7	11 15 63 202 204 232 240	36 93 97 98 106 115 160	31 31 68 76 89 106 162	16 23 63 222 204 221 231	38 93 106 111 123 115 169	31 38 77 87 90 110 156
	10	48 49 55 55 79 96 103 122 176 232	30 63 110 141 148 152 178 208 232 239	100 121 147 149 154 161 209 213 224 241	30 39 55 63 79 96 109 136 196 243	36 66 111 136 152 165 188 208 230 234	89 123 147 145 162 169 211 216 232 246
Test 6	2	156 172	76 136	42 127	156 173	75 136	42 128
	5	29 48 78 92 180	38 73 138 145 221	60 78 153 202 236	23 38 81 106 176	36 71 132 139 214	54 74 146 211 232
	7	36 115 128 130 176 203 211	37 71 89 151 155 163 189	53 85 170 209 225 232 254	31 120 136 140 181 201 221	39 64 84 146 152 169 191	46 76 168 203 219 229 242
	10	16 35 53 61 64 73 75 196 211 226	64 95 125 133 145 192 198 215 218 238	32 35 53 81 149 170 171 224 228 240	13 38 55 69 74 79 89 203 216 236	58 88 118 146 150 192 203 219 222 240	31 38 55 78 151 168 176 226 239 245
Test 7	2	123 160	92 109	108 199	122 161	92 110	107 199
	5	25 96 177 193 230	82 97 133 149 220	21 82 116 153 209	31 102 179 200 234	80 103 128 145 218	19 78 111 146 203
	7	23 34 34 46 113 142 231	68 137 139 146 149 164 250	19 28 79 113 118 156 159	19 33 35 42 111 138 230	66 132 133 144 148 162 243	23 35 77 111 121 155 162
	10	61 63 79 110 115 132 150 162 181 189	59 83 91 106 109 113 116 163 189 225	12 45 51 83 136 177 189 186 217 220	57 62 80 111 116 134 152 160 179 192	60 84 90 111 116 119 121 152 184 221	11 41 48 78 126 163 179 192 213 224
Test 8	2	89 142	53 211	86 177	88 141	51 142	87 177
	5	54 142 178 228 245	76 108 200 248 251	68 86 91 106 132	52 138 176 223 239	71 99 189 239 248	67 83 89 111 128
	7	43 43 64 92 108 141 153	42 108 134 172 173 184 245	16 29 129 138 143 192 215	38 45 66 92 111 141 155	42 111 128 172 181 192 234	19 36 129 146 155 191 211
	10	14 34 63 106 137 171 187 203 212 241	26 43 111 158 173 196 213 223 233 239	39 48 66 94 142 158 161 190 206 209	11 33 62 102 133 164 181 212 216 237	31 43 105 146 177 196 219 223 240 249	28 44 66 100 144 162 169 201 211 235

**Table A2:** Comparison of the *p*-value and H-value obtained by the Wilcoxon signed-rank test between the pairs of mSAR vs LFD, mSAR vs HHO, mSAR vs SCA, mSAR vs EO, mSAR vs GSA, and mSAR vs AOA for fitness outcomes using Fuzzy Entropy’s method.

		LFD		HHO		SCA		EO		GSA		AOA		SAR	
Test Image	nTh	P	H	P	H	P	H	P	H	P	H	P	H	P	H
Test 1	2	6.0277E-13	1	6.1441E-07	1	1.3234E-06	1	NaN	0	3.5515E-05	1	1.8168E-04	1	3.2683E-07	1
	5	2.8784E-14	1	2.9055E-01	0	1.0896E-10	1	NaN	0	6.6496E-05	1	NaN	0	NaN	0
	7	7.6340E-01	0	7.7059E-11	1	1.1971E-01	0	1.5134E-14	1	6.3305E-01	0	NaN	0	3.6698E-01	0
	10	6.0867E-16	1	3.4468E-08	1	1.3333E-16	1	1.3664E-14	1	1.5773E-07	1	2.8047E-10	1	NaN	0
Test 2	2	2.8843E-14	1	3.4468E-01	0	3.4228E-11	1	NaN	0	8.7150E-10	1	1.5045E-03	1	NaN	0
	5	3.7658E-07	1	NaN	0	6.0880E-06	1	1.3284E-01	0	8.9271E-13	1	1.7487E-11	1	1.6951E-01	0
	7	7.1903E-01	0	1.8322E-06	1	6.3259E-09	1	1.6634E-14	1	2.8648E-01	0	8.7888E-05	1	2.1346E-01	0
	10	2.8784E-14	1	9.6534E-08	1	NaN	0	1.4164E-14	1	6.2125E-15	1	2.0439E-05	1	1.5679E-07	1
Test 3	2	NaN	0	NaN	0	1.9354E-11	1	NaN	0	3.7768E-07	1	2.2483E-07	1	1.5843E-04	1
	5	2.7230E-01	0	2.1070E-12	1	1.9354E-06	1	1.3808E-01	0	2.1030E-01	0	5.5186E-12	1	1.4070E-08	1
	7	8.3963E-15	1	2.3017E-10	1	5.3941E-01	0	1.1664E-14	1	8.6481E-07	1	9.8562E-15	1	3.3336E-01	0
	10	3.0718E-13	1	9.7777E-08	1	1.4390E-09	1	1.4315E-14	1	1.1266E-01	0	8.3233E-14	1	3.3336E-01	0
Test 4	2	3.8096E-08	1	9.3557E-01	0	5.9317E-01	0	1.5765E-14	1	6.2876E-01	0	NaN	0	NaN	0
	5	3.7658E-06	1	3.8424E-12	1	1.0573E-06	1	1.3664E-01	0	3.9056E-08	1	4.0878E-07	1	NaN	0
	7	4.0161E-10	1	1.7406E-10	1	1.3620E-01	0	1.3646E-15	1	8.1116E-05	1	3.3498E-03	1	NaN	0
	10	6.5873E-16	1	9.4370E-09	1	1.3333E-16	1	1.6634E-15	1	1.8348E-10	1	1.6465E-15	1	1.4069E-01	0
Test 5	2	NaN	0	NaN	0	NaN	0	NaN	0	2.3713E-06	1	NaN	0	NaN	0
	5	8.8756E-06	1	2.5657E-10	1	3.9604E-10	1	NaN	0	2.2425E-10	1	4.4022E-06	1	1.9822E-01	0
	7	4.5622E-01	0	1.9040E-10	1	2.6701E-10	1	3.1557E-05	1	6.3412E-01	0	1.1344E-08	1	1.8464E-01	0
	10	2.8784E-01	0	3.8776E-01	0	3.7095E-13	1	1.3019E-15	1	8.7232E-12	1	1.7373E-15	1	1.8024E-06	1
Test 6	2	2.8603E-01	0	1.1192E-01	0	8.8169E-01	0	2.1092E-14	1	1.0730E-10	1	2.3619E-10	1	2.3411E-01	0
	5	2.7077E-09	1	1.1192E-14	1	8.8169E-10	1	2.1092E-01	0	1.0730E-07	1	2.4459E-01	0	3.2202E-01	0
	7	6.1778E-15	1	8.0918E-16	1	2.1505E-14	1	NaN	0	4.5494E-01	0	2.3408E-12	1	2.5595E-01	0
	10	1.7293E-16	1	8.6143E-08	1	2.7239E-18	1	NaN	0	1.1159E-01	0	6.4539E-01	0	2.5546E-01	0
Test 7	2	2.8653E-08	1	3.9472E-01	0	2.8673E-01	0	1.5765E-14	1	6.2876E-07	1	1.7835E-05	1	2.5459E-01	0
	5	2.7077E-09	1	3.8428E-06	1	1.3512E-06	1	1.0111E-05	1	2.3713E-03	1	9.6307E-03	1	2.8447E-05	1
	7	1.2173E-12	1	1.5557E-13	1	4.7489E-15	1	1.4366E-14	1	1.1052E-08	1	9.3744E-08	1	3.2304E-06	1
	10	2.9808E-13	1	2.0897E-01	0	1.5107E-15	1	1.6634E-01	0	9.0344E-01	0	2.7644E-12	1	NaN	0
Test 8	2	3.9119E-10	1	3.8428E-08	1	NaN	0	NaN	0	5.7833E-03	1	NaN	0	NaN	0
	5	3.9484E-05	1	3.8428E-05	1	NaN	0	1.4624E-14	1	8.0258E-06	1	NaN	0	NaN	0
	7	2.6925E-07	1	1.8575E-01	0	1.2903E-01	0	NaN	0	6.3841E-01	0	1.0330E-01	0	3.3336E-01	0
	10	1.7407E-01	0	1.0298E-13	1	4.5518E-16	1	1.4318E-15	1	1.5987E-15	1	NaN	0	NaN	0

**Table A3:** The optimal threshold values are obtained by the Otsu method.

		mSAR			SAR		
Test Image	nTh	Red	Green	Blue	Red	Green	Blue
Test 1	2	70 167	79 194	101 215	72 170	88 192	71 170
	5	215 136 153 182 182	82 116 137 182 232	47 112 128 150 209	215 133 150 182 120	82 120 141 182 230	50 110 131 150 210
	7	71 93 127 174 180 190 212	74 85 85 152 180 245 254	78 95 117 141 201 217 232	73 92 165 174 183 190 212	73 85 78 150 180 240 254	78 98 110 117 201 217 230
	10	65 77 132 134 135 162 177 184 198 255	75 84 97 129 158 159 179 188 197 218	87 102 103 107 135 151 231 238 246 250	68 77 123 134 135 160 177 184 200 246	80 89 96 129 160 159 179 190 197 220	87 102 103 110 136 151 216 236 246 253
Test 2	2	63 155	75 115	154 165	65 145	73 118	150 162
	5	127 135 136 143 226	75 112 145 172 207	20 98 141 197 210	130 135 142 151 230	76 139 148 182 210	46 105 160 200 213
	7	90 126 135 176 180 183 213	88 90 120 127 145 162 183	70 91 120 127 145 161 182	92 121 142 180 186 187 218	86 94 117 122 147 167 186	73 94 127 130 144 167 188
	10	31 60 73 82 96 105 136 151 224 230	13 28 71 79 89 108 148 185 224 251	19 23 23 85 97 129 135 178 190 207	33 63 76 83 98 104 133 151 226 234	14 28 73 79 88 108 164 185 228 254	23 27 23 87 97 129 137 178 196 210
Test 3	2	40 174	15 106	15 123	38 170	18 109	13 128
	5	28 112 117 145 189	73 87 94 126 191	43 60 79 80 153	29 112 117 140 195	72 90 95 130 194	43 58 96 103 169
	7	23 48 96 133 213 217 217	17 82 86 93 143 175 213	13 29 48 68 83 113 119	24 49 101 145 177 200 216	35 37 46 186 188 196 216	19 36 56 123 163 192 230
	10	33 44 59 60 65 143 147 192 239 245	64 67 68 70 86 142 145 162 180 223	60 73 98 112 119 132 155 158 176 239	35 60 60 78 102 146 196 200 210 245	64 69 70 90 99 104 168 189 200 229	63 71 76 98 165 173 190 209 230 243
Test 4	2	39 176	39 142	49 155	43 177	40 144	53 156
	5	28 116 120 145 189	101 114 121 156 223	44 61 77 82 154	25 120 149 163 203	100 123 136 160 241	38 56 68 93 159
	7	62 73 104 117 119 135 198	40 49 130 142 145 186 250	60 85 88 100 173 179 234	63 109 132 140 189 206 233	43 53 120 156 190 206 230	68 77 89 106 163 176 204
	10	10 16 48 73 78 138 147 150 239 245	46 60 67 73 119 176 179 219 169 215	58 73 98 112 119 132 155 158 223 258	13 19 49 76 79 145 149 180 201 246	42 56 86 146 159 169 180 210 233 243	60 73 90 101 120 136 163 180 206 239
Test 5	2	40 109	36 86	80 148	46 110	37 84	78 148
	5	24 68 73 160 179	33 39 159 161 183	63 111 128 149 220	26 71 83 86 183	30 40 160 168 194	68 100 130 145 218
	7	29 41 68 89 98 108 143	46 58 103 106 169 209 246	89 99 100 113 126 240 254	32 41 76 85 93 110 144	40 55 102 100 163 210 241	90 103 110 123 180 235 239
	10	53 66 69 76 87 126 196 210 214 223	29 55 63 79 106 173 179 196 199 226	93 106 116 117 120 148 153 163 242 242	55 66 70 80 91 112 196 215 220 223	34 58 63 75 110 173 177 196 200 229	92 110 116 120 123 156 163 166 249 250
Test 6	2	39 176	41 142	48 155	41 179	43 156	49 159
	5	32 112 120 148 187	103 115 123 158 223	43 59 79 82 153	34 113 123 186 193	108 132 142 169 230	45 63 82 86 160
	7	83 105 129 143 170 181 238	103 115 129 198 203 230 233	23 80 110 116 136 190 230	86 100 124 140 163 178 235	102 113 132 200 206 241 245	25 79 112 119 143 201 248
	10	15 66 69 76 87 126 196 210 214 223	35 73 83 96 129 148 160 200 241 242	15 46 64 69 158 167 189 189 211 232	18 62 71 80 91 130 200 214 216 220	37 70 79 87 123 152 172 206 230 254	18 53 57 79 146 163 182 184 210 229
Test 7	2	70 94	109 126	88 140	73 96	112 132	87 143
	5	49 76 165 205 205	81 86 128 162 210	24 69 133 139 220	42 80 168 201 206	84 93 134 160 215	25 70 130 142 217
	7	65 92 129 138 149 163 216	30 86 104 130 131 132 205	16 86 104 120 139 145 198	62 89 124 135 152 164 210	29 84 102 129 138 136 209	18 89 100 118 141 149 201
	10	56 58 76 126 135 162 183 186 221 238	34 46 76 79 130 149 172 189 236 252	54 62 64 64 141 142 158 198 228 253	58 60 81 130 137 164 189 189 222 240	30 41 71 80 129 151 170 184 232 248	55 64 69 88 146 152 162 203 230 249
Test 8	2	63 203	32 131	43 149	68 205	34 130	46 142
	5	31 116 121 143 189	73 86 93 134 196	45 62 81 83 155	28 113 136 145 193	76 92 99 124 201	52 65 95 90 193
	7	83 95 125 143 139 152 215	107 120 136 198 204 234 240	49 57 83 109 136 198 230	81 94 119 148 169 170 229	104 116 120 190 208 228 243	53 63 89 117 140 212 229
	10	29 68 95 99 117 128 148 176 221 238	49 68 104 112 119 129 158 163 236 252	38 47 49 56 94 99 112 149 228 253	31 65 93 106 120 139 143 172 220 249	56 87 100 111 139 142 179 184 210 238	32 54 55 68 103 123 130 168 180 241

**Table A4:** Comparison of the *p*-value and H-value obtained by the Wilcoxon signed-rank test between the pairs of mSAR vs LFD, mSAR vs HHO, mSAR vs SCA, mSAR vs EO, mSAR vs GSA, and mSAR vs AOA for fitness outcomes using the Otsu method.

		LFD		HHO		SCA		EO		GSA		AOA		SAR	
Test Image	nTh	P	H	P	H	P	H	P	H	P	H	P	H	P	H
Test 1	2	5.4624E-10	1	5.5908E-01	0	1.2044E-01	0	NaN	0	3.4391E-03	1	1.6672E-01	0	2.9745E-01	0
	5	2.6084E-11	1	2.6439E-01	0	9.9165E-06	1	1.5636E-13	1	6.4391E-01	0	2.2570E-06	1	3.0340E-07	1
	7	6.9180E-10	1	7.0120E-06	1	1.0895E-08	1	1.5306E-13	1	6.1301E-02	0	7.6170E-03	1	3.3399E-01	0
	10	5.5159E-13	1	3.1364E-01	0	1.2135E-11	1	1.5346E-13	1	1.5273E-05	1	2.5737E-09	1	NaN	0
Test 2	2	2.6137E-11	1	3.1364E-01	0	3.1152E-06	1	1.5300E-01	0	8.4391E-01	0	1.3807E-01	0	1.5125E-01	0
	5	3.4126E-01	0	1.5526E-03	1	5.5409E-01	0	NaN	0	8.6445E-11	1	1.6047E-10	1	1.5427E-01	0
	7	6.5159E-06	1	NaN	0	5.7574E-04	1	NaN	0	2.7741E-10	1	8.0651E-04	1	NaN	0
	10	2.6084E-11	1	8.7841E-03	1	3.0826E-12	1	1.5346E-13	1	6.0158E-13	1	1.8756E-04	1	1.4270E-01	0
Test 3	2	8.2306E-01	0	5.4887E-01	0	1.7615E-06	1	1.4586E-01	0	3.6573E-05	1	2.0632E-05	1	1.4419E-01	0
	5	NaN	0	1.9173E-07	1	1.7615E-01	0	NaN	0	2.0364E-06	1	5.0641E-10	1	NaN	0
	7	7.6088E-12	1	2.0944E-05	1	4.9093E-01	0	NaN	0	8.3743E-02	0	9.0446E-13	1	NaN	0
	10	2.7837E-10	1	8.8971E-01	0	1.3097E-04	1	1.5606E-13	1	1.0910E-06	1	7.6379E-12	1	NaN	0
Test 4	2	3.4523E-01	0	8.5131E-01	0	5.3986E-01	0	1.4790E-13	1	6.0885E-01	0	3.8450E-07	1	1.5925E-01	0
	5	3.4126E-01	0	3.4964E-05	1	9.6229E-02	0	1.8626E-13	1	3.7820E-06	1	3.7512E-05	1	1.6804E-01	0
	7	3.6394E-07	1	1.5838E-05	1	1.2396E-04	1	1.5631E-13	1	7.8548E-03	1	3.0739E-01	0	1.8804E-01	0
	10	5.9695E-13	1	8.5872E-04	1	1.2135E-11	1	1.5606E-13	1	1.7767E-08	1	1.5109E-13	1	1.2804E-01	0
Test 5	2	7.0424E-01	0	8.7424E-01	0	3.6045E-01	0	1.5300E+00	0	2.2962E-01	0	7.0856E-01	0	1.6475E-01	0
	5	8.0431E-01	0	2.3346E-05	1	3.6045E-05	1	NaN	0	2.1715E-08	1	3.7825E-01	0	NaN	0
	7	4.1343E-07	1	1.7325E-05	1	2.4302E-05	1	NaN	0	6.1405E-02	0	1.0410E-06	1	1.6804E-01	0
	10	2.6084E-11	1	3.5284E-06	1	3.3762E-08	1	1.5316E-13	1	8.4471E-10	1	1.5943E-13	1	1.6404E-01	0
Test 6	2	2.5921E-01	0	1.0184E-09	1	8.0245E-05	1	1.9788E-12	1	1.0390E-05	1	2.1674E-08	1	2.1307E-01	0
	5	2.4538E-06	1	1.0184E-09	1	8.0245E-05	1	1.9788E-12	1	1.0390E-05	1	2.1674E-08	1	2.9307E-01	0
	7	5.5983E-12	1	7.3631E-11	1	1.9572E-09	1	1.5306E-13	1	4.4054E-01	0	2.1882E-11	1	2.3294E-01	0
	10	1.5671E-13	1	7.8385E-01	0	2.4791E-13	1	1.3645E-13	1	1.0806E-06	1	NaN	0	2.3249E-01	0
Test 7	2	NaN	0	NaN	0	2.6096E-01	0	NaN	0	NaN	0	1.6672E-01	0	NaN	0
	5	2.4538E-06	1	3.4967E-01	0	1.2298E-01	0	9.4860E-02	0	2.2962E-01	0	9.0029E-02	0	2.4494E-01	0
	7	1.1032E-09	1	1.4156E-08	1	4.3222E-10	1	1.5366E-13	1	1.0702E-06	1	8.7632E-07	1	NaN	0
	10	2.7012E-10	1	1.9015E-05	1	1.3749E-10	1	1.5066E-13	1	8.7484E-11	1	2.5842E-11	1	NaN	0
Test 8	2	3.5450E-01	0	3.4967E-01	0	9.4924E-02	0	NaN	0	5.6002E-01	0	7.7421E-01	0	NaN	0
	5	3.5781E-01	0	3.4967E-01	0	5.7411E-05	1	1.5346E-13	1	7.7717E-02	0	5.6581E-04	1	4.6219E-05	1
	7	NaN	0	1.6902E-01	0	1.1743E-01	0	NaN	0	6.1821E-06	1	NaN	0	3.0340E-06	1
	10	NaN	0	9.3703E-09	1	4.1427E-11	1	NaN	0	1.5481E-13	1	7.6170E-12	1	3.9900E-01	0
